# Radiosynthesis and characterization of [^18^F]BS224: a next-generation TSPO PET ligand insensitive to the rs6971 polymorphism

**DOI:** 10.1007/s00259-021-05617-4

**Published:** 2021-11-16

**Authors:** Sang Hee Lee, Nunzio Denora, Valentino Laquintana, Giuseppe Felice Mangiatordi, Angela Lopedota, Antonio Lopalco, Annalisa Cutrignelli, Massimo Franco, Pietro Delre, In Ho Song, Hye Won Kim, Su Bin Kim, Hyun Soo Park, Kyungmin Kim, Seok-Yong Lee, Hyewon Youn, Byung Chul Lee, Sang Eun Kim

**Affiliations:** 1 Department of Nuclear Medicine, Seoul National University Bundang Hospital, Seoul National University College of Medicine, Seongnam, 13620 Republic of Korea; 2 Department of Transdisciplinary Studies, Graduate School of Convergence Science and Technology, Seoul National University, Seoul, 08826 Republic of Korea; 3 Department of Pharmacy – Drug Sciences, University of Bari “A. Moro”, 70121 Bari, Italy; 4 Institute of Crystallography, National Research Council, Via G. Amendola 122/O, 70126 Bari, Italy; 5 Department of Chemistry, University of Bari “A. Moro”, Via E. Orabona, 4, 70125 Bari, Italy; 6 Department of Nuclear Medicine, Seoul National University Hospital, Seoul, 03080 Republic of Korea; 7 Department of Biomedical Sciences, Seoul National University Graduate School, Seoul, 03080 Republic of Korea; 8 Laboratory of Molecular Imaging and Therapy, Cancer Research Institute, Seoul National University College of Medicine, Seoul, 03080 Republic of Korea; 9 Center for Nanomolecular Imaging and Innovative Drug Development, Advanced Institutes of Convergence Technology, Suwon, 16229 Republic of Korea; 10 Department of Molecular Medicine and Biopharmaceutical Sciences, Graduate School of Convergence Science and Technology, Seoul National University, Seoul, 08826 Republic of Korea

**Keywords:** Translocator protein 18-kDa, BS224, Aromatic ^18^F-fluorination, Neuroinflammation, rs6971 polymorphism

## Abstract

**Purpose:**

Translocator protein 18-kDa (TSPO) positron emission tomography (PET) is a valuable tool to detect neuroinflammed areas in a broad spectrum of neurodegenerative diseases. However, the clinical application of second-generation TSPO ligands as biomarkers is limited because of the presence of human rs6971 polymorphism that affects their binding. Here, we describe the ability of a new TSPO ligand, [^18^F]BS224, to identify abnormal TSPO expression in neuroinflammation independent of the rs6971 polymorphism.

**Methods:**

An in vitro competitive inhibition assay of BS224 was conducted with [^3^H]PK 11195 using membrane proteins isolated from 293FT cells expressing TSPO-wild type (WT) or TSPO-mutant A147T (Mut), corresponding to a high-affinity binder (HAB) and low-affinity binder (LAB), respectively. Molecular docking was performed to investigate the interaction of BS224 with the binding sites of rat TSPO-WT and TSPO-Mut. We synthesized a new ^18^F-labeled imidazopyridine acetamide ([^18^F]BS224) using boronic acid pinacol ester **6** or iodotoluene tosylate precursor **7**, respectively, via aromatic ^18^F-fluorination. Dynamic PET scanning was performed up to 90 min after the injection of [^18^F]BS224 to healthy mice, and PET imaging data were obtained to estimate its absorbed doses in organs. To evaluate in vivo TSPO-specific uptake of [^18^F]BS224, lipopolysaccharide (LPS)-induced inflammatory and ischemic stroke rat models were used.

**Results:**

BS224 exhibited a high affinity (*K*_i_ = 0.51 nM) and selectivity for TSPO. The ratio of IC_50_ values of BS224 for LAB to that for HAB indicated that the TSPO binding affinity of BS224 has low binding sensitivity to the rs6971 polymorphism and it was comparable to that of PK 11195, which is not sensitive to the polymorphism. Docking simulations showed that the binding mode of BS224 is not affected by the A147T mutation and consequently supported the observed in vitro selectivity of [^18^F]BS224 regardless of polymorphisms. With optimal radiochemical yield (39 ± 6.8%, decay-corrected) and purity (> 99%), [^18^F]BS224 provided a clear visible image of the inflammatory lesion with a high signal-to-background ratio in both animal models (BP_ND_ = 1.43 ± 0.17 and 1.57 ± 0.37 in the LPS-induced inflammatory and ischemic stroke rat models, respectively) without skull uptake.

**Conclusion:**

Our results suggest that [^18^F]BS224 may be a promising TSPO ligand to gauge neuroinflammatory disease-related areas in a broad range of patients irrespective of the common rs6971 polymorphism.

**Supplementary Information:**

The online version contains supplementary material available at 10.1007/s00259-021-05617-4.

## Introduction

The translocator protein 18-kDa (TSPO) is expressed predominantly in steroidogenic tissues [1]. In the central nervous system (CNS), an abnormal overexpression of TSPO is generally observed in activated microglia in patients with neurodegenerative disorders such as Alzheimer’s disease, Parkinson’s disease, Huntington’s disease, and multiple sclerosis [2]. In vivo brain imaging of activated microglia using positron emission tomography (PET) could detect pathological inflammatory states, characterized by the death and loss of neurons, which are consequences of neuronal dysfunctions. Thus, the development of specific TSPO PET ligands may help monitor the progression of pathologies associated with inflammatory responses and help assess the efficacy of specific therapies designed to control CNS diseases [3].

**Fig. 1:**
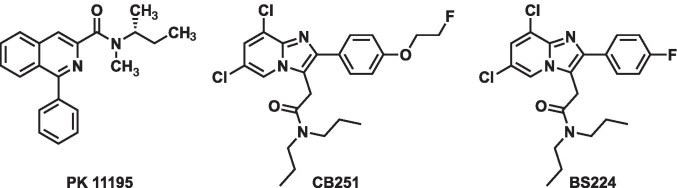
Structure of PK 11195, CB251, and BS224

Several research groups have focused on introducing fluorine-18 (^18^F) or carbon-11 (^11^C) into different structural classes of chemical compounds, such as isoquinoline carboxamides (e.g., PK 11195) [4], aryloxyanilides (e.g., PBR28 and fm-PBR28-*d*_2_) [5, 6], and pyrazolopyrimidine acetamides (e.g., DPA-713 and DPA-714) [7, 8]. In the last decade, numerous second-generation TSPO ligands have been developed, and some of them have exhibited higher specific to non-specific signals than the first-generation TSPO ligand, PK 11195. However, these ligands display binding differences that depend on a single nucleotide polymorphism (rs6971), which involves the substitution of alanine with threonine at position 147 (A147T) in the fifth transmembrane domain of the TSPO gene [9]. Interpretation of PET results of second-generation TSPO ligands is not straightforward, as clinical trial participants may have a high-affinity binder (HAB) or low-affinity binder (LAB) phenotype depending on their particular TSPO gene polymorphism and TSPO binding features. Most second-generation TSPO ligands display a desirable high-affinity for TSPO visualization in the HAB phenotype, but exhibit poor TSPO affinity in the LAB phenotype [10]. This variation in ligand-binding affinity complicates the quantitative assessment of PET data. Furthermore, the poor affinity results in lower signal-to-background ratio in PET imaging in individuals with the LAB phenotype. Consequently, the clinical usefulness of second-generation TSPO ligands is limited in patients with the LAB phenotype compared with that in patients with the HAB phenotype [11]. Curiously, the loss of binding affinity of first-generation TSPO ligand, [^11^C]PK 11195 is not dependent on the rs6971 polymorphism [12]. However, its clinical use is still limited due to reported disadvantages such as high non-specific binding, high plasma protein binding, low blood–brain barrier permeability, and short half-life of ^11^C [13]. Therefore, many studies have focused on developing new TSPO ligands to overcome the clinical issues of second-generation TSPO ligands caused by TSPO polymorphism. In this respect, previously, we reported that the imidazo[1,2-a]pyridine scaffold shows a high affinity and selectivity for TSPO, and we prepared its ^18^F-labeled form [^18^F]CB251 as a new TSPO ligand [14, 15]. Recently, we demonstrated that [^18^F]CB251 has a great potential in assessing neuroinflammation as it displays higher TSPO affinity and selectivity, regardless of the rs6971 polymorphism phenotype, compared with previously described TSPO ligands [16]. However, [^18^F]CB251 PET showed an unspecific uptake in the skull in our preclinical studies due to defluorination of the parent molecule. This skull image will hinder the exact quantification of TSPO expression in the clinical brain images, because signal can also be detected in the adjacent brain regions such as the cortex, due to a spillover effect [17]. Therefore, we designed an aromatic ^18^F-labeled imidazo[1,2-a]pyridine analog, named [^18^F]BS224, as it was expected to have the same biological properties as [^18^F]CB251 due to the molecular similarity between fluorine and fluoroethanol (Fig. 1). [^18^F]BS224 could endure in vivo defluorination because the aromatic C-F bond is stronger than the aliphatic C-F bond [18]. In this study, we evaluated which of the two precursors should be preferred for the direct aromatic ^18^F-fluorination of [^18^F]BS224. Furthermore, to the best of our knowledge, we present the first biological characterization of [^18^F]BS224 demonstrating that it is a new promising TSPO ligand and that its binding is not dependent on rs6971 polymorphism.

## Materials and method

### Chemistry

The detailed experimental procedures for the synthesis of the authentic compound (**1**, BS224) and precursors (**6** and **7**) are described in the experimental section in the Supplemental Information (SI). Briefly, two halogen-substituted imidazo[1,2-a]pyridines (**1** and **4**) were synthesized in three steps from 3-(4-fluorobenzoyl)propionic acid or 3-(4-bromobenzoyl)propionic acid. Dipropyl amides (**2a**-**b**) were prepared through amide-coupling reaction, followed by bromination to generate two bromine substituted benzoyl compounds (**3a**-**b**). BS224 (**1**) and its bromine substituted intermediate **4** used as precursors can be readily prepared directly from **3a**-**b** and 2-amino-3,5-dichloropyridine. In order to prepare precursors (**6** and **7**), the trimethylstannane compound (**5**) was prepared from **4** in the presence of bis(trimethyl)tin and the catalytic quantity of tetrakis(triphenylphosphine)palladium, followed by exposure to 4-(diacetoxy)iodotoluene to obtain **7**. Using the same bromo compound **4**, we synthesized a different type of precursor, boronic acid pinacol ester **6**. The ^18^F-labeled form, [^18^F]BS224, was successfully synthesized using nucleophilic aromatic substitution (S_N_Ar) ^18^F-labeling method with boronic acid pinacol ester **6** or iodotoluene tosylate precursor **7**.

### Radiosynthesis of 2-(-2-(4-[^18^F]fluorophenyl)-6,8-dichloro-imidazo[1,2-a]pyridin-3-yl)-N,N-dipropylacetamide ([^18^F]BS224)

Radiochemistry of [^18^F]BS224 was performed using a well-established aromatic ^18^F-fluorination method, with slight modifications [19, 20]. In order to introduce fluorine-18 in the distal phenyl group in the imidazopyridine acetamide analog, we conducted S_N_Ar ^18^F-labeling of **6** or **7**. Briefly, 50–100 µL of ^18^F (18.5–37 MBq) in ^18^O-enriched water was diluted with deionized water (900–950 µL) and passed through a Chromafix-HCO_3_ cartridge (pre-activated with 2 mL of ethanol and 5 mL of water). The captured ^18^F ion on the cartridge was eluted with a mixture solution of 18-Crown-6 (0.18 mg for **6** or 3.5 mg for **7**) dissolved in 1 mL of methanol, and CsHCO_3_ (0.10 mg for **6** or 1.1 mg for **7**) in 10 µL of water. The eluted mixture with ^18^F was dried by azeotropic distillation with acetonitrile (CH_3_CN) twice (0.3 mL × 2) at 90 °C under a stream of nitrogen gas. A solvent mixture with **6** (3.0 mg, 5.7 μmol), Cu(OTf)_2_ (1.2 mg, 3.4 μmol), and pyridine (8.2 μL, 102 μmol) was added to the reaction vial containing the dried 18-Crown-6/^18^F^−^Cs^+^ complex (18.5–1110 MBq), followed by heating at 110 °C for 10 min.

For precursor **7**, the isolation of ^18^F from ^18^O-enriched water was obtained by following the same method used for precursor **6**. The residue mixture was dissolved in the desired solvent and then transferred in to a 4 mL vial containing precursor **7** (4.4. mg, 5.7 μmol) and 2,2,6,6-tetramethyl-1-piperidinyloxy (TEMPO, 1 mg, 6.4 μmol), followed by heating at different temperatures for 10 min. For both ^18^F-labeling methods, we followed the same purification step. After cooling in an ice-bath, the reaction mixture was diluted with aqueous 0.1 N HCl (10 mL). This solution was loaded into a C18 plus Sep-Pak cartridge, washed with water (10 mL), and eluted with CH_3_CN (1.0 mL). After dilution with 1.5 mL of water, the solution components were separated using a semi-preparative HPLC system (Waters, Xterra semi-preparative C18 column, 10 mm × 250 mm, 10 µm; 55% CH_3_CN-water containing 0.1% formic acid; *λ* = 254 nm, flow rate = 3.0 mLmin^−1^) equipped with a UV detector and a gamma-ray detector. The fraction of [^18^F]BS224 (*T*_R_ = 28 min) collected from the HPLC system was diluted with water (20 mL). The HPLC solution was exchanged with 10% ethanol-saline solution using a C18 plus Sep-Pak cartridge for the preclinical studies.

### Log D measurement

The lipophilicity (log D) of [^18^F]BS224 was measured five times by mixing a solution of [^18^F]BS224 (7.4 MBq) in 5% ethanol-saline (10 μL) with phosphate-buffered saline (PBS, 5.0 mL, 0.15 M, pH 7.4) and n-octanol (5 mL) in a test tube. After vortexing for 1 min, each tube was placed on a table at 25 °C for 3 min. Samples of each phase (100 μL) were counted for radioactivity using a gamma-counter. Log D is expressed as the logarithm of the ratio of the count of n-octanol to that of PBS.

### *In vitro *stability of [^18^F]BS224

To assess in vitro serum stability, [^18^F]BS224 in 5% ethanol-saline (3.7 MBq, 0.2 mL) was incubated with human serum (1.8 mL) from a healthy volunteer for 120 min at 37 °C. Aliquots (0.2 mL) were collected at 0, 15, 30, 60, and 120 min and mixed with acetonitrile (0.2 mL), and then centrifuged at 3500 rpm for 5 min. The resulting supernatants were analyzed using a radio-TLC scanner with the developing solvent (50% ethyl acetate-hexane).

### Plasma protein binding measurement

Plasma protein binding (PPB) was measured using two plasma samples obtained from five rats and a healthy volunteer. Plasma-free fraction (*f*_p_) of [^18^F]BS224 was estimated using an ultrafiltration method [21]. [^18^F]BS224 (0.37 MBq) in 5% ethanol/saline (10 µL) was added to each plasma samples (*n* = 5 for rat or human, 300 µL) and saline (*n* = 5, 300 µL), and then the samples were incubated at 37 °C for 10 min. The mixtures were transferred to the Amicon® Ultra 30 K device (Merck Millipore, USA), and centrifuged at 12,000 rpm for 15 min. The radioactivity of [^18^F]BS224 before (AP_total_ for plasma, AS_total_ for control) and after filtration (AP_free_ for plasma, AS_free_ for control) was measured using a gamma-counter. The plasma-free fraction (*f*_P_) was calculated as *f*_p_ = (AP_free_ /AP_total_)/(AS_free_/AS_total_).

### *In vivo *radiometabolic stability of [^18^F]BS224

In vivo metabolic stability test was performed using male Sprague–Dawley rats (7–8 weeks old, Orientbio Inc.). [^18^F]BS224 (37 MBq) was intravenously injected into the rat tail vein. The rats were sacrificed by carbon dioxide inhalation at 30 and 90 min (*n* = 3 for each time point) after [^18^F]BS224 injection. The tissues (brain, kidneys, lung, heart, femur, and liver) were collected and homogenized with 1:1.4 v/v PBS (pH 7.4) and CH_3_CN (2 mL), and then centrifuged at 3000 rpm for 15 min at 4 °C. Blood (3 mL) was collected into a Li-heparin tube and then centrifuged at 3000 rpm for 15 min at 4 °C. After centrifugation, the supernatant (1 mL) was collected and mixed with CH_3_CN (1.4 mL). After centrifuging again, the supernatant was analyzed using a semi-preparative HPLC system (Waters, Xterra semi-preparative C18 column, 10 mm × 250 mm, 10 µm; 65% CH_3_CN-water containing 0.1% formic acid; *λ* = 254 nm, flow rate = 3.0 mLmin^−1^). The percentages of [^18^F]BS224 (*T*_R_ = 14.5 min) to the total radioactivity (corrected for decay) on the HPLC chromatogram was calculated as % = (peak area for [^18^F]BS224/total peak area) × 100.

### *In vitro* binding assay

The in vitro binding affinity of BS224 was performed via the displacement of [^3^H]PK 11195 and [^3^H]flunitrazepam binding from rat cerebral cortex samples; the result is expressed as inhibition constant (*K*_i_). The binding assay was performed following a previously published procedure [22], with slight modifications.

### Competitive inhibition assay of TSPO ligands in HAB or LAB phenotype

The effect of the rs6971 polymorphism on the binding affinity of BS224 was evaluated using an in vitro competitive inhibition assay (IC_50_). Briefly, the IC_50_ value of BS224 (using different concentrations ranging from 0.1 nM to 1000 μM) was measured based on competition with [^3^H]PK 11195 (0.019 MBq/100 μL) in membrane proteins (100 μg/100 μL) that were isolated from 293FT cells expressing TSPO-WT or TSPO-Mut, which represented the HAB or LAB phenotypes, respectively.

### Homology modelling and molecular docking simulations

Following to a previously reported method [23], we built homology models of rat TSPO-WT and rat TSPO-Mut based on the published solution structures of mouse TSPO-WT [24] and mouse TSPO-Mut [25], respectively. The models obtained was employed to perform docking simulations for BS224 and PK 11195 (see methodological details in the SI).

### Animals for PET imaging studies

Normal male C57BL/6 mice (*n* = 4, 6–7 weeks old, Orientbio Inc.) were used to perform in vivo organ distribution and dosimetry studies (see methodological details in the SI). Male Sprague–Dawley rats (7–8 weeks old, Orientbio Inc.) were used to create rat models of neuroinflammation and ischemic stroke (*n* = 4 for each model). An acute neuroinflammation rat model was generated by injecting lipopolysaccharides (LPS, 50 μg) into the right striatum of rat brain according to our previous protocol [26]. An ischemic stroke rat model was generated via 60-min occlusion of the right middle cerebral artery (MCAO), as previously described [27]. In the case of LPS-induced neuroinflammatory model, rats were anesthetized with ketamine hydrochloride (50 mg/kg) and xylazine hydrochloride (0.2 mg/kg) before LPS injection. In the case of ischemic stroke model, rats were anesthetized with 5% isoflurane in the presence of 30% nitrous oxide and 70% oxygen, and then were under anesthesia with 2% isoflurane in the presence of 30% nitrous oxide and 70% oxygen during MCAO surgery.

### Animal PET imaging studies in LPS-induced neuroinflammatory and ischemic stroke rat models

The PET imaging study was performed using a dedicated animal PET/CT scanner (NanoPET/CT; Mediso Inc., Budapest, Hungary) with a 10-cm axial field-of-view (FOV) and a 12-cm transaxial FOV. Before PET imaging, the rats were anesthetized with 2% isoflurane. LPS-induced neuroinflammatory and ischemic stroke rat models (*n* = 4 for each model) were used for PET imaging at 4-day post-LPS injection and 11-day post-MCAO surgery, respectively. Brain PET images were obtained for 90 min with 54 frames (12 × 10 s, 16 × 30 s, 8 × 60 s, and 18 × 240 s) immediately after the intravenous injection of [^18^F]BS224 (30.2 ± 1.6 MBq), followed by a CT scan. All data were reconstructed into three-dimensional images using the iterative 3D ordered subset expectation maximization (OSEM) algorithm to generate dynamic PET images.

### PET imaging analysis

The volumes of interest (VOIs) were drawn around the peak PET activity on the ipsilateral region in a coronal PET/CT slice, and symmetrically pasted into the contralateral side on the same slice to yield a contralateral VOI of identical volume and shape. Time-activity curves (TACs) for ipsilateral and contralateral region were generated. Decay-corrected radioactivity in the VOI was expressed as the standardized uptake value (SUV), which was normalized to the injected radioactivity and body weight of the rat using PMOD software version 3.6 (PMOD Technologies Ltd., Zurich, Switzerland). The area under curve (AUC) was calculated as the trapezoidal sum of the observed data over the range of 0–90 min. The binding potential (BP_ND_) was also calculated as a quantitative value, which represented the receptor binding with the radioligand. The BP_ND_ was estimated using a simplified reference-tissue model (SRTM) by taking the TAC of the contralateral side as the reference region [28].

### Histologic and Immunofluorescent Staining

Histologic staining and immunofluorescence studies were carried out on the ischemic stroke rats after the PET imaging. After PET imaging, the ischemic stroke rats were sacrificed by carbon dioxide inhalation. The rat brain was immediately extracted. Each brain was cut into 2-mm coronal sections and immersed in 2% solution of triphenyltetrazolium chloride (TTC) in saline at 37 °C for 30 min. After removing the TTC solution, the brain sections were then fixed with 10% formalin and then photographed. In contrast, the immunofluorescent staining was conducted on the rat brain tissue that had undergone PET imaging to evaluate TSPO and microglia expression in the ischemic brain region. The brain was transcardially perfused with 10% formalin. The isolated brains were embedded in a freezing section compound, frozen, and consecutively cryosectioned in 10-μm thickness slices using a cryocut microtome (Leica Biosystems). The brain sections were stained with rabbit anti-TSPO antibody or rat anti-CD11b antibody according to the manufacturer’s instructions. After washing with PBS for three times, these sections were incubated with Alexa 488-conjugated goat anti-rabbit IgG or Alexa 546-conjugated goat anti-rat IgG. All the antibodies were purchased from Thermo Fisher Scientific. The slides were mounted with SlowFade Gold antifade reagent with DAPI. Fluorescent images were acquired using a confocal microscope (LSM 710, Zeiss).

### Statistical analysis

Values were expressed as mean ± standard deviations. Statistical analysis was performed using GraphPad Prism software (version 7.0, GraphPad Software Inc.). *P* < 0.05 was considered statistically significant. The binding and competitive inhibition assay data were analyzed using GraphPad Prism 7.0 software (GraphPad Software Inc., CA, USA). The curves were fitted using a one site binding model with a sum-of-squares *F* test for *K*_i_ and paired Student’s *t*-test for IC_50_ (*P* value was less than 0.05), respectively. The specific binding was determined by subtracting non-specific binding (maximal concentrations of BS224), which was normalized to 100% specific binding (no BS224).

## Results

### Radiochemistry

**Fig. 2:**
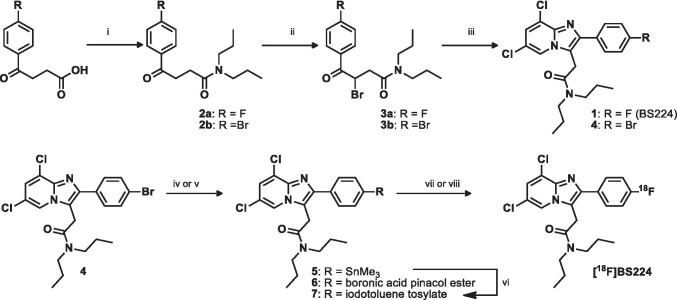
Synthesis of precursors (**6** & **7**), synthesis of BS224 (**1**), and radiosynthesis of [^18^F]BS224. Conditions: (i) *N*,*N’*-dipropylamine, CDI, Et_3_N, anhydrous THF, 25 °C, 4 h; (ii) Br_2_, CCl_4_, 25 °C, 2 h; (iii) 2-amino-3,5-dichloropyridine, DMF, reflux, 18 h; (iv) Sn_2_Me_6_, Pd(PPh_3_)_4_, dioxane, 105 °C, 6 h; (v) Pd(dppf)Cl_2_, CH_3_COOK, B_2_pin_2_, DMF, 80 °C, 3 h; (vi) TsOH, 4-(diacetoxy)iodotoluene, CH_3_CN-CHCl_3_, 50 °C, 18 h; (vii) **6**, 18-Crown-6, Cs^18^F Cu(OTf)_2_, pyridine, DMF, 110 °C, 10 min; (viii) **7**, 18-Crown-6, Cs^18^F, TEMPO, CH_3_CN, 140 °C, 10 min

We synthesized two precursors as shown in Fig. 2 and performed aromatic ^18^F-labeling under various experimental conditions to prepare [^18^F]BS224 (Table 1). Using a phase-transfer catalyst/base complex (18-Crown-6/CsHCO_3_), the ^18^F-labeling of **6** was carried out via copper-mediated ^18^F-fluorination. The use of tetrakis(pyridine)copper(II) triflate catalyst for aromatic ^18^F-fluorination failed to yield [^18^F]BS224, but its individual agents (i.e., COpper(II) triflate in the presence of pyridine) produced the desired product with 9.3% yield (entries 1 and 2). When a considerably lower amount of the 18-Crown-6 (0.7 μmol)/CsHCO_3_ (0.5 μmol) complex was used, the yield of [^18^F]BS224 increased linearly (up to 65%) depending on the solvent (entries 3–5). In contrast, higher amounts of Cu(OTf)_2_ and pyridine (Entry 6) did not increase the radiochemical yield (RCY) of [^18^F]BS224 compared with their optimal amounts (3.3 μmol of Cu(OTf)_2_ and 0.1 mmol of pyridine). The synthesis of [^18^F]BS224 using **7** in *N*,*N*-dimethylformaide (DMF) or CH_3_CN solvent at 100 ℃ resulted in a poor RCY of the final product (entries 7 and 8). These low yields of [^18^F]BS224 were easily overcome when the reaction temperature was increased up to 140 ℃ with CH_3_CN in a pressure tube (entry 10). The radioactivity of ^18^F for the optimization of the reaction condition showed in Table 1 ranged from 18.5 to 37 MBq. In in vivo studies, the radio-synthetic procedure of [^18^F]BS224 using **6** included pre-purification on a Sep-Pak cartridge and purification via HPLC and resulted in 39 ± 6.8% (*n* = 8, decay corrected) RCY and with high radiochemical purity (> 99%, See Fig. S2 and S3 in the SI). [^18^F]BS224 was formulated and ready for use in preclinical PET imaging studies with a 10% ethanol-saline solution. The *A*_M_ of [^18^F]BS224 starting with ^18^F (740–1110 MBq) ranged from 315 to 472 GBq/µmol. The identity of [^18^F]BS224 was confirmed by co-injection with the authentic compound **1** using analytical HPLC (See Fig. S4 in the SI).

**Table 1:** Radiosynthesis of [^18^F]BS224 using two different precursors (**6**^a^ or **7**^b^) under various conditions

Entry	Additive	Solvent	*T* (°C)	RCY(%)^*c*^
1^d^	Cu(Py)_4_(OTf)_2_	DMF	110	N.R
2^d^	Cu(OTf)_2_ + pyridine	DMF	110	9 ± 3
3	Cu(OTf)_2_ + pyridine	DEF	110	22 ± 3
4	Cu(OTf)_2_ + pyridine	DMA	110	45 ± 4
5	Cu(OTf)_2_ + pyridine	DMF	110	65 ± 9^*e*^
6^f^	Cu(OTf)_2_ + pyridine	DMF	110	26 ± 2
7	TEMPO	DMF	100	N.R
8	TEMPO	CH_3_CN	100	4 ± 2
9	TEMPO	CH_3_CN	120	18 ± 5
10	TEMPO	CH_3_CN	140	26 ± 4

The schematic illustration for ^18^F-labeling of [^18^F]BS224 is shown in Fig. 2. *N.R.* no reaction, *RCY* radiochemical yield

^a^The ^18^F-labeling condition (entries 1–6): **6** (3 mg, 5.7 μmol), 18-Crown-6 (0.18 mg, 0.7 μmol), CsHCO_3_ (0.1 mg, 0.5 μmol), Cu(OTf)_2_ (1.2 mg, 3.3 μmol), and pyridine (8 μL, 0.1 mmol) in solvent (0.3 mL) at 110 °C for 10 min (*n* = 3)

^b^The ^18^F-labeling condition (entries 7–10): **7** (4.4 mg, 5.7 μmol), 18-Crown-6 (3.5 mg, 13.3 μmol), CsHCO_3_ (1.1 mg, 5.7 μmol), and TEMPO (1 mg, 6.4 μmol) in solvent (0.3 mL) at different temperatures (*T*) for 10 min (*n* = 5)

^c^Radiochemical yield was measured by radio-TLC (See Fig. S2)

^d^18-Crown-6 (3.5 mg) and CsHCO_3_ (1.1 mg)

^e^*n* = 18

^f^Cu(OTf)_2_ (3.6 mg, 10.0 μmol) and pyridine (24 μL, 0.3 mmol)

### Measurement of Log D, metabolic stability, and plasma protein binding

The partition coefficient value (Log D) of [^18^F]BS224 was 2.78 ± 0.04 (See Table S1 in the SI). The in vitro serum stability of [^18^F]BS224 showed that over 99% of the parent compound remained intact for 120 min (See Fig. S5 in the SI). In addition, in vivo radiometabolite assay in healthy rats showed that [^18^F]BS224 is highly stable in the brain, heart, kidneys, and lung at both 30 and 90 min, and a single peak corresponding to the parent compound was observed for these organs (Table 2). In contrast, two major polar metabolites were detected in both liver and plasma. In liver, a relatively higher metabolism was observed (73.6% and 53.1% of the parent fraction, respectively, at 30 and 90 min after [^18^F]BS224 injection) compared with in the plasma (See Fig. S8 in the SI). The plasma free fraction (*f*_p_) of [^18^F]BS224 was 6.4 ± 0.3 and 4.9 ± 0.5% in rat and human, respectively (See Table S2 in the SI).

**Table 2:** Percentage of radiometabolites and unmetabolized parent of [^18^F]BS224 in the plasma and organs

Time (min)	% in brain		% in heart		% in kidneys	
	Metabolites	Parent	Metabolites	Parent	Metabolites	Parent
30	–	Intact	–	Intact	–	Intact
90	–	Intact	–	Intact	–	Intact
Time (min)	% in lung		% in liver		% in plasma	
	Metabolites	Parent	Metabolites	Parent	Metabolites	Parent
30	–	Intact	26.4 ± 5.9	73.6 ± 5.9	9.3 ± 0.8	90.7 ± 0.8
90	–	Intact	46.9 ± 2.7	53.1 ± 2.7	30.8 ± 2.9	69.2 ± 2.9

^a^Data are presented as Mean ± SD. Three healthy male Sprague–Dawley rats were used for each time point

### K_i_ and IC_50_ values of BS224 in the HAB or LAB phenotype

As shown in Table 3, BS224 showed a high binding affinity to TSPO (*K*_i_ = 0.51 ± 0.03 nM) and a high selectivity to central benzodiazepine receptor (CBR, *K*_i_ > 10^5^), which were similar to the values observed for CB251. This result indicated that BS224 preserves the biological characteristic of CB251 without the molecular dissimilarity. In addition, the IC_50_ of LAB/IC_50_ of HAB for BS224, which represents a measure of different affinities of the ligand to different TSPO genotypes, was 0.76, which is similar to that for CB251. All curves for *K*_i_ and IC_50_ are shown in Fig. S6 in the SI.

**Table 3:** *K*_i_ and IC_50_ values of TSPO Ligands

Ligand	*K*_i_ (nM)^a^		IC_50_ (μM)^b^			
	TSPO	CBR		HAB	LAB	Ratio of LAB/HAB
BS224	0.51 ± 0.03^c^	> 10^5^		0.59 ± 0.04	0.45 ± 0.02	0.76
CB251	0.27 ± 0.09^c^	> 10^5^		0.27 ± 0.01	0.31 ± 0.02	1.14
PK 11195	1.74 ± 0.41^c^ (1.38 ± 0.42)^c^	> 10^5^		6.05 ± 0.75	5.01 ± 0.10	0.83
fmPBR28-*d*_2_	3.68 ± 0.70^c^	> 10^5^		0.20 ± 0.01	7.61 ± 0.01	37.28
Flunitrazepam^d^	–	6.2 ± 0.1		–	–	–

^a^The in vitro binding affinities (*K*_i_) of ligands were measured by displacement of [^3^H]PK 11195 or [^3^H]flunitrazepam from rat cerebral cortex samples

^b^Competitive inhibition assay with TSPO ligands in the presence of [^3^H]PK 11195 was performed in membrane proteins isolated 293FT cells expressing polymorphic TSPOs. HAB indicates high affinity ligand-binding phenotype (TSPO-WT). LAB indicates low-affinity ligand-binding phenotype (TSPO-Mut) IC_50_ values of BS224, CB251, PK 11195, and fmPBR28-*d*_2_ was obtained simultaneously in the sample experiment as described in a reference[16]

^c^*K*_i_ values of CB251 and PK 11195 (in parentheses) are according to references[6, 14]

^d^The CBR selective ligand was used for comparison. Data are means ± SEM of three separate experiments performed in duplicate

### Molecular docking simulations

**Fig. 3:**
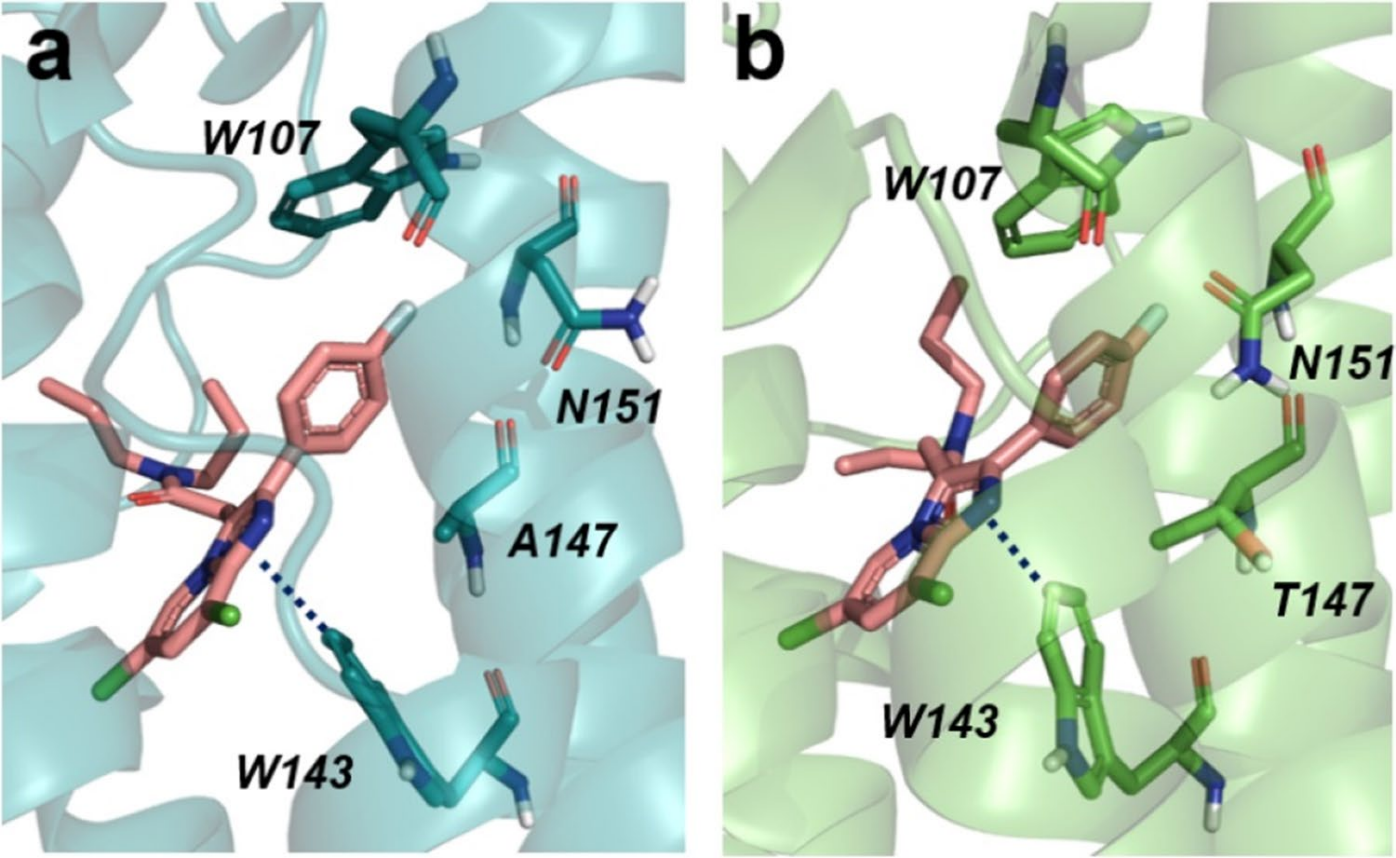
Top-scored docking poses of BS224 in rat TSPO-WT (**a**); BS224 in rat TSPO-Mut (**b**). Proteins are rendered as cartoons while BS224 and important residues are rendered as sticks. For the sake of clarity, only polar hydrogens are shown. Dotted blue lines indicate *π*-*π* interactions

The A147T substitution in mouse TSPO is responsible for a restricted conformational effect involving only a few residues in proximity of the mutation and belonging to the binding site. This is evident by comparing the solution structures of TSPO-WT and TSPO-Mut. In other words, an in-depth investigation of the effect of such localized structural modification on ligand binding is necessary to shed light on the reasons behind the TSPO-WT selectivity exhibited by many TSPO ligands. Based on this evidence, and with the aim of providing a molecular rationale for the observed experimental data, we carried out molecular docking simulations on the homology models of wild type rat TSPO-WT and rat TSPO-Mut using Glide, available from the Schrödinger suite as software. Figure 3 shows the top-scored docking poses of BS224 in both rat TSPO-WT and rat TSPO-Mut. Consistent with the experimental data, the binding mode observed when inspecting the solution structures of both mouse TSPO-WT and mouse TSPO-Mut in complex with the reference ligand PK 11195 is herein fully confirmed: molecular recognition is mostly due to a T-shaped *π*-*π* interaction with W143 and hydrophobic interactions with several residues belonging to the binding pocket. Furthermore, as the evident in Fig. 3, the binding is not affected by the A147T mutation. In particular, the para-fluoro phenyl group is oriented towards the cytosolic side and is accommodated by a small sub-pocket consisting of W107 and N151. The obtained docking scores strongly support the robustness of the predicted binding model. Indeed, consistent with the experimental data, the docking scores obtained for BS224 within both rat TSPO-WT (− 10.157 kcal/mol) and rat TSPO-Mut (− 8.175 kcal/mol) were similar to those resulting from the calibration of the NMR cognate ligand PK 11195, being equal to − 10.090 kcal/mol and − 9.896 kcal/mol for rat TSPO-WT and rat TSPO-Mut, respectively.

### Dosimetry

Regional time-activity curves of organs showed that [^18^F]BS224 was highly accumulated in the peripheral organs such as the heart, lung, kidneys, and liver in healthy mice at early time. Low *C*_max_ and AUC values of the bladder, compared with those of the kidneys, suggested that [^18^F]BS224 was not excreted into the bladder via the urine (See Fig. S7 and Table S3 in the SI). The liver showed the highest estimated human absorbed dose, 28.88 ± 7.70 μGy/MBq after the [^18^F]BS224 injection. The kidneys, heart, and adrenals also showed higher absorbed doses. The estimated whole-body human effective dose was 5.18 µSv/MBq.

### PET studies in LPS-induced neuroinflammation and ischemic stroke rat models

**Fig. 4:**
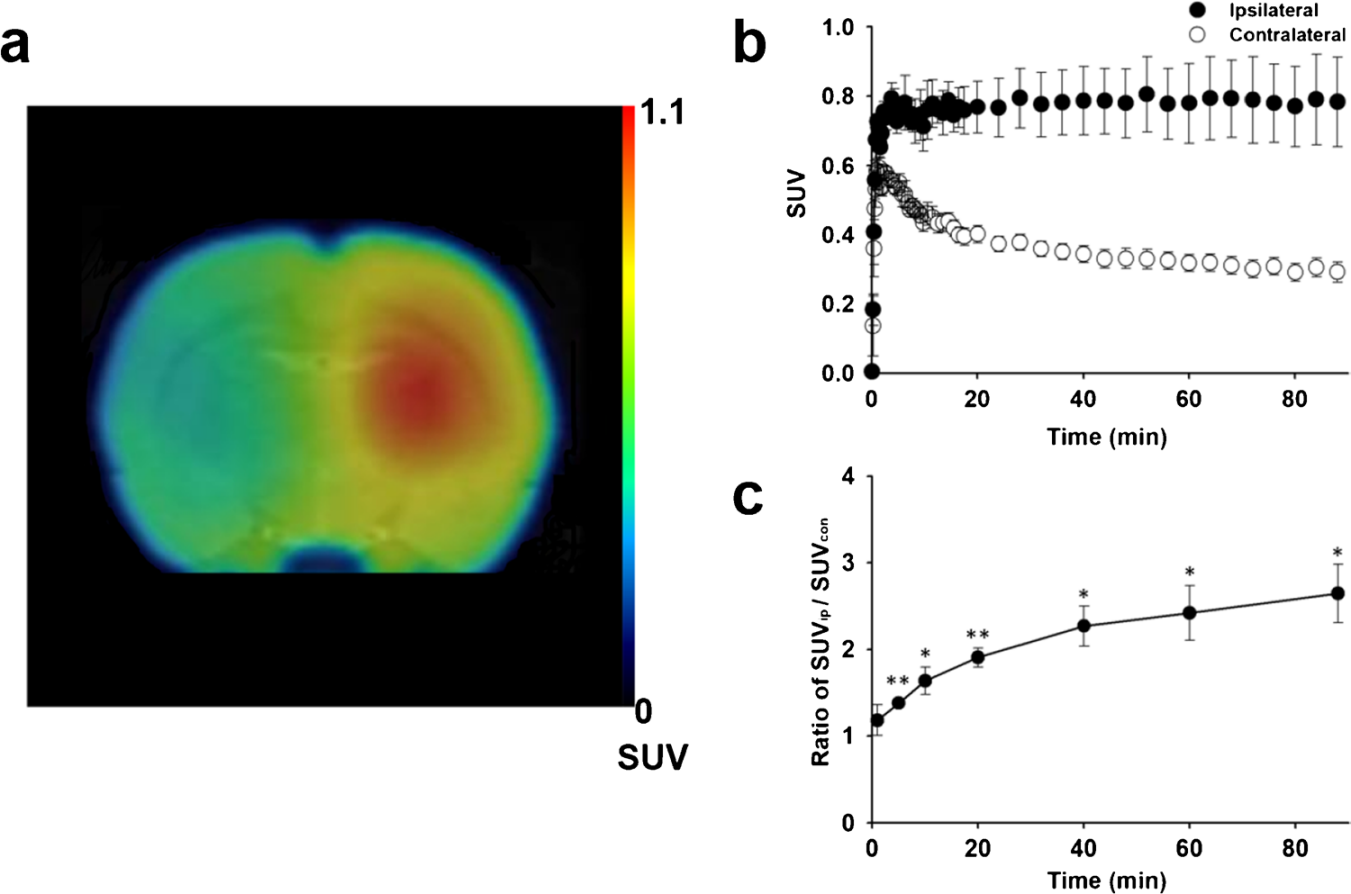
Representative fused PET/MR image (**a**) and SUV time-activity curves (**b**) of [^18^F]BS224. The ratio of SUV of the ipsilateral side to that of the contralateral side (**c**) at each time point (1, 5, 10, 20, 40, 60, and 90 min) in the LPS-induced neuroinflammatory rat model. ROI was masked by MRI template. Ipsilateral means LPS-injected brain area. Contralateral means the opposite of ipsilateral. *P < 0.05, **P < 0.01

**Fig. 5:**
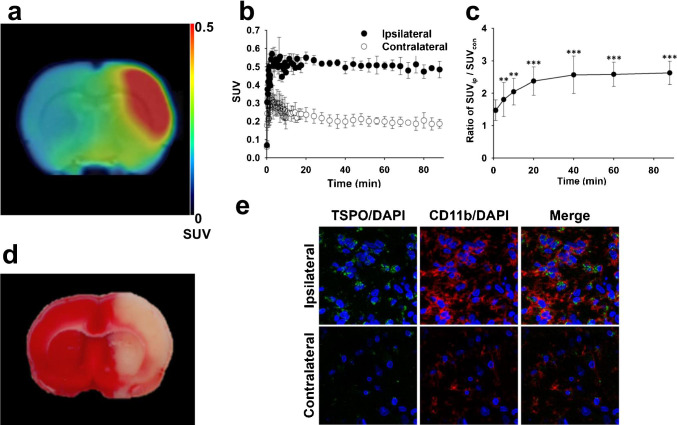
Representive fused PET/MR image (**a**) and SUV time-activity curves (**b**) of [^18^F]BS224. The ratio of SUV of the ipsilateral side to that of the contralateral side (**c**) at each time point (1, 5, 10, 20, 40, 60, and 90 min) in the ischemic stroke rat model. TTC staining (**d**) and the immunohistochemical staining (**e**) of the brain slices. ROI was masked by MRI template. Fluorescence images indicates the expression of TSPO and microglia following the development of lesions. Blue color: DAPI, green: TSPO, red: CD11b, Scale bars, 10 μm (× 40). **P < 0.01; ***P < 0.001

[^18^F]BS224 accumulated at high levels in the right ipsilateral striatum in the LPS-induced inflammation rat model during PET imaging (Fig. 4a). The time-course of [^18^F]BS224 distribution also graphically demonstrated the uptake and clearance of [^18^F]BS224 between the ipsilateral and contralateral regions (Fig. 4b). A high accumulation of [^18^F]BS224 was observed in the ipsilateral (the maximum concentration; *C*_max_ = 0.945 ± 0.178 SUV, the time of maximum concentration; *T*_max_ = 3.9 min) and contralateral (*C*_max_ = 0.732 ± 0.159 SUV, *T*_max_ = 1.4 min) regions early during imaging. During the late phases of imaging, especially for accumulated [^18^F]BS224, its rapid wash-out was observed in the contralateral region. Finally as shown Fig. 4c, the uptake ratio of [^18^F]BS224 in the ipsilateral region to that in the contralateral region was 2.646 ± 0.336 (*P* = 0.0318) at 90 min. The AUC value of the ipsilateral region was 2.15-folds higher (*P* = 0.0208) (AUC = 68.03 ± 9.42) than that of the contralateral region (AUC = 31.68 ± 2.76). BP_ND_ of [^18^F]BS224 was 1.43 ± 0.17. Next, PET studies of [^18^F]BS224 were also performed in the ischemic stroke rat model (Fig. 5a), and we successfully visualized ischemia lesions with a high signal-to-background ratio (BP_ND_ = 1.57 ± 0.37). Similar to the results in the LPS-induced neuroinflammatory rat model, a high accumulation of [^18^F]BS224 was observed in the ipsilateral (*C*_max_ = 0.569 ± 0.030 SUV, *T*_max_ = 2.7 min) and the contralateral region (*C*_max_ = 0.327 ± 0.049 SUV, *T*_max_ = 0.6 min) at early during imaging, followed by rapid wash-out in the contralateral region (Fig. 5b). In contrast, [^18^F]BS224 accumulated in the ipsilatral region was sustained during end of the imaging. The maximum uptake ratio of [^18^F]BS224 in the ipsilateral side to the contralateral side was 2.629 ± 0.359 (*P* = 0.0006) at 90 min (Fig. 5c). The AUC value of the ipsilateral (AUC = 44.53 ± 1.72) was 2.36-folds higher (*P* = 0.0002) than that of the contralateral region (AUC = 18.84 ± 2.79) in the ischemic stroke rat model. The results obtained from the PET images of [^18^F]BS224 were consistent with the TTC staining result (Fig. 5d). TTC effectively delineates infarcted areas appearing devoid of red staining. Both TSPO and microglia expression were assessed by immunohistochemical staining of the ischemic area in the rat brain, as represented in the co-registered [^18^F]BS224 PET images and TTC stained brain sections. Compared with the contralateral side, the ipsilateral ischemic lesion showed higher expression of TSPO in the microglia with intense CD11b immunoreactivity (Fig. 5e). The autoradiography results confirmed the selectivity and specificity of previously performed TSPO PET results, with no inhibition and significant reduction of radioactivity in flumazenil- and PK 11195-treated ischemic stroke rat brain slices, respectively (See Fig. S9 in the SI). These results demonstrate that ischemia is also well reflected by [^18^F]BS224 binding in PET imaging. In addition, the skull uptake was not observed in either of the rat models.

## Discussion

Recently, several ^18^F-labeled TSPO radiotracers with different molecular structures which were insensitive to the rs6971 genetic polymorphism have been reported (e.g., [^18^F]FEBMP, (*R*,*S*)-[^18^F]GE387, (*R*)-[^18^F]NEBIFQUINIDE, [^18^F]LW223, and [^18^F]PBR316). One of these is an acetamidobenzoazolone analog; [^18^F]FEBMP was evaluated for PET imaging of neuroinflammation in rat and in vitro autoradiography on postmortem human brains. In the in vitro autoradiographic assay of human brain tissues, [^18^F]FEBMP showed a LAB/HAB ratio of 0.9. TSPO rs6971 polymorphism appeared to have a little influence on the binding sites for [^18^F]FEBMP, but this radioligand showed a metabolic stability not eligible in rats as its radiolabeled metabolite rapidly increased in the plasma and its was also found in the brain tissue [29]. (*R*,*S*)-[^18^F]GE387 overcame the binding sensitivity of the tricyclic ligand [^18^F]GE180; however, further investigation of each enantiomer for brain imaging remained [30]. Other ligands, (*R*)-[^18^F]NEBIFQUINIDE and [^18^F]LW223, two examples of ^18^F-labeled analog of PK11195, presented insensitivity to the rs6971 polymorphism, but PET imaging of the neuroinflammatory model has not been performed [31, 32]. [^18^F]PBR316 showed a nanomolar affinity for TSPO, and low sensitivity to rs6971 polymorphism with a LAB/HAB ratio of 1.5. The metabolite analysis showed that the principal radioactivity was in the brain (and adrenal glands), owing to the original radiotracer [33]. In this study, we report the high metabolic stability in the brain and insensitive to the rs6971 polymorphism, and visualization in vivo preclinical brain inflammatory of the novel aromatic ^18^F-labelled 2-phenyl-imidazo[1,2-a]pyridine analog [^18^F]BS224 as a new-generation TSPO PET ligand for neuroinflammation imaging.

Regarding the procedure for aromatic ^18^F-fluorination on the right phenyl of BS224, the electrophilic ^18^F-labeling method is not suitable for a TSPO PET ligand because of a low molar activity (*A*_M_) of the final product. Furthermore, this imidazo[1,2-a]pyridine substituent at the para position would not provide sufficient activation for the incorporation of ^18^F using conventional S_N_Ar ^18^F-labeling methods [34]. There have been several efforts to develop new S_N_Ar ^18^F-labeling precursors such as hypervalent iodines [35], organoborons [36], and organostannanes [37], to obtain radiotracer with improved radiochemical yield as well as in vivo metabolic stability. For these reasons, we designed two different types of precursors to attain a feasible S_N_Ar ^18^F-labeling strategy to overcome the radio-synthetic problem of [^18^F]BS224. Based on well-established aromatic ^18^F-labeling methods for fluoroarenes including those described in our previous studies [19, 20], we selected two different types of precursors (**6** and **7)** and compared their potency on aromatic ^18^F-fluorination of [^18^F]BS224, as shown in Table 1. In the case of **6**, individual addition with Cu(II) triflate and excess pyridine was more efficient for the aromatic ^18^F-fluorination of [^18^F]BS224 than Cu(Py)_4_(OTf)_2_. In addition, the highest ^18^F-fluorination efficiency was obtained when DMF and a lower amount of 18-Crown-6/CsHCO_3_ were used. These increased ^18^F-fluorination effects might be due to the additive pyridine that has a stabilizing effect on the complex of metal ^18^F in the presence of minimum amounts of 18-Crown-6/CsHCO_3_ [38]. In the reaction using precursor **7**, the RCY was improved by changing the solvent from DMF to CH_3_CN and also by increasing the temperature. Therefore, precursor **6** required relatively low temperatures in the preparation of [^18^F]BS224 and produced approximately 2.5-fold higher RCY than that obtained under the final optimized conditions for precursor **7**. Indeed, even though a reasonable RCY was achieved using **7** as the precursor, precursor **6** was considerably superior in terms of yield and *A*_M_ of [^18^F]BS224. The partition coefficient (log D = 2.78 ± 0.04) value of [^18^F]BS224 was close to that of [^11^C]PBR28 (log D = 2.82 ± 0.07) among the aryloxyanilide derivatives [6]. According to the results of a previous study [39], aromatic ^18^F-labeled form, [^18^F]F-DPA was shown much higher metabolic stability than aliphatic ^18^F-labeled form, [^18^F]DPA-714 which suffers *O*-deethylation in vivo environment in the rat brain. In our study, aromatic ^18^F-labeled form, [^18^F]BS224 also showed high in vitro serum stability and in vivo metabolic stability in rat organs, except in the liver and plasma at 90 min. In particular, we measured the radioactivity in the femur by gamma counting to estimate in vivo defluorination of [^18^F]BS224 in this experiment, but, unlike that in other organs, no radioactivity could be detected in the femurs. Our results suggested that [^18^F]BS224 has high chemical stability due to fluorine-18 attached to the aryl ring to combat defluorination and no polar radiometabolites are expected to cross the blood–brain barrier. Both values (*f*_p_ = 6.4 ± 0.3% in rat and 4.9 ± 0.5% in human) indicate that [^18^F]BS224 has a relatively lower %PPB value than that reported for [^11^C]PK 11195 (*f*_p_ = 1% in human) [40]. In addition, these *f*_p_ values of [^18^F]BS224 were comparable with the reported *f*_p_ values of other second-generation TSPO ligands (e.g., [^18^F]F-DPA, 6.6% in rat; [^18^F]AB5186, 6.7% in rat; [^18^F]PBR111, 5–7% in human; [^11^C]PBR28, 4.1% in human; [^18^F]GE-180, 3.5% in human) [39, 41–43].

The in vitro experiments performed with membrane proteins isolated from 293FT cells and molecular docking simulations allowed us to evaluate the binding capacity of the radiotracer [^18^F]BS224 and explain its low sensitivity to the rs6971 polymorphism. The main advantage of this approaches is that it is easier to secure the polymorphic target proteins (A147A and A147T) when compared with human blood and tissues samples generally used to isolate polymorphic TSPOs. Here, we developed a cell system using human embryonic kidney cell lines (293FT) which ectopically express TSPO A147A and A147T leading to higher yield of polymorphic proteins. These cells also allow us to overcome the limitation of having to recruit human volunteers who express the rs6971 polymorphism. Indeed, only 4% of Japanese and 2% of Han Chinese are reported to express the rs6971 polymorphism in the now retired HapMap Resource (http://hapmap.ncbi.nlm.nih.gov), so we could assume that it would be extremely difficult to recruit LAB donors within less than 3% of Korean population to test the in vitro sensitivity of BS224 to the rs6971 polymorphism.

As reported previously [44, 45], we used membrane proteins isolated from human embryonic kidney 293FT cells expressing polymorphic TSPOs (WT for HAB or mutant A147T for LAB) for the competitive inhibition assay of BS224 and PK 11195. This binding assay using polymorphic TSPOs from genetically modified human cells can provide the evaluation for the polymorphism of TSPO without using human-derived tissues or blood in places where human samples cannot be obtained.

As results, the obtained IC_50_ ratio of LAB/HAB for PK 11195 was close to the reported LAB/HAB ratio obtained from human blood and brain tissue [12, 31, 46]. In particular, in vitro cell binding of [^18^F]BS224 between LAB and HAB showed the favorable insensitivity to the rs6971 polymorphism. Although our system may overcome the difficulties of in vitro screening using human samples, evaluation of [^18^F]BS224 in humans should be performed in subjects with polymorphic TSPO phenotype as a further study.

We also proved that the binding affinity of [^18^F]BS224 for TSPO is not affected by the rs6971 polymorphism, in agreement with the molecular docking simulations. The performed docking simulations complemented the experimental findings suggesting a molecular rationale for the observed low sensitivity of BS224 to the rs6971 polymorphism. Importantly, an in-depth visual inspection of the developed homology models clearly showed that, upon mutation, N151 is subjected to a conformational rearrangement that place its side chain within the binding site (Fig. 3). Of note, such a different orientation is evident also when comparing the solution structures used as templates. In other words, we can reasonably postulate, based on the top-scored docking structures obtained from the simulation, that the presence of a bulky para-phenyl substituent may be responsible for a higher sensitivity to the rs6971 polymorphism, as its accommodation within the TSPO binding site can be sterically hindered by the N151 conformational rearrangement occurring upon mutation. In addition, we presented the comparable docking scores of BS224 and PK 11195 using molecular docking simulations on the homology models of wild type rat TSPO-WT and rat TSPO-Mut. Human TSPO sequences around A147, which is involved in the rs6971 polymorphism, are similar between species. Especially, amino acid sequences between 130 and 148 (VSPLAARLLYLAWLAFAT) are exactly the same in mouse, rat, and human [24]. Therefore, molecular docking analysis in TSPO-WT and TSPO-mut (A147T) using rat TSPO represents polymorphic differences in human. Although in silico data cannot explain the physiological environment of animals and human, we believe that in silico data can be useful in screening ligand candidates for clinical translation [23, 47].

Extrapolated human effective dose of [^18^F]BS224 was 5.18 µSv/MBq and this level was closed to that of [^11^C]PK 11195 estimated in rodent (4.2 µSv/MBq) [48] which was comparable with its reported effective dose level (4.8 µSv/MBq) in healthy human [49]. In addition, this preclinical level of [^18^F]BS224 is expected to be lower than that of [^18^F]DPA-714 (17.2 µSv/MBq) in healthy human [50] and will be having the similar level with other TSPO ligands such as [^11^C]PBR-28 (6.6 µSv/MBq) and [^11^C]DPA-713 (5.9 µSv/MBq) [51, 52]. Dosimetry results based on whole-body PET imaging of mice could be suggested as a preclinical tool for the preliminary estimation of the absorbed and effective doses before a clinical study [53, 54].

As described in previous studies, high TSPO expression was observed in the LPS-induced neuroinflammatory rat model [55] 4 days after injection and was maximum 11 days following ischemic stroke [56, 57]. Therefore, [^18^F]BS224 TSPO PET imaging was conducted at 4 day post-LPS injection and 11 day post-MCAO surgery. Preclinical in vivo images of [^18^F]BS224 in rat models demonstrated that [^18^F]BS224 provided a clear visualization of inflammatory lesions compared with that in the contralateral sides. Based on reported results, [^18^F]BS224 shows relatively high levels of BP_ND_ compare with [^11^C]PK 11195 in both models [28, 58]. In addition, the BP_ND_ value (1.43 ± 0.17) of [^18^F]BS224 in LPS-induced neuroinflammatory rat models is comparable to those of other second-generation TSPO ligands such as [^11^C]PBR28 and [^18^F]DPA-714 [14, 58]. Compared with our previous results, BP_ND_ of the aliphatic ^18^F-labeled imidazolpyridine analogue [^18^F]CB251 was slightly higher than that of the aromatic ^18^F-labeled imidazolpyridine analogue [^18^F]BS224 in the same model which was produced in the same laboratory. The BP_ND_ value of [^18^F]CB251 was 1.83 ± 0.18, which is comparable with that of [^11^C]PBR28 (BP_ND_ 1.55 ± 0.41) in PET imaging study using the same animals. However, [^18^F]CB251 PET exhibited an unspecific uptake in the skull due to defluorination in vivo environment. For this reason, we designed and prepared the aromatic ^18^F-labeled form, [^18^F]BS224 in this study. In case of [^18^F]GE-180, the BP_ND_ seems to be relatively high in the LPS-induced inflammatory and ischemic stroke rat models [58, 59]; however [^18^F]GE-180 was suffering from issues. One is the binding sensitivity to the rs6971 polymorphism (*K*_i_ ratio of LAB/HAB = 5.1) and another is low BBB penetration [60].

Over the years, various quantification approaches and methods for parameter estimation have been used for the kinetic modeling of TSPO PET in preclinical and clinical studies [61, 62]. Full compartmental modeling with arterial input function (AIF) and two-tissue compartment model (2TCM) is recognized as the gold standard for quantifying TSPO PET radioligands. Besides, recent simplifications in modeling such as a standardized uptake value ratio (SUVr), simplified reference-tissue model (SRTM), and supervised clustering approach (SVCA) using anatomical or extracted reference regions have been suggested as alternative quantification methods to the full compartmental modeling. These simplified methods provided reliable quantification of TSPO expression without AIF in the modeling [63]. Among them, SRTM is one of the most used quantification methods in the early stage of developing of radioligand candidates using unilateral inflammatory animal models such as LPS-induced neuroinflammation and MCAO rat model [55, 56], because the contralateral region can be used as a reference region instead of invasive approaches like blood sampling. In this study, we evaluated the in vivo characteristics of [^18^F]BS224 in two different inflammatory animal models using the SRTM approach to provide a comparison the BP_ND_ of [^18^F]BS224 with our previous results. However, TSPO is constitutively expressed in the brain and therefore the use of the contralateral side as reference region for the ipsilateral side might not be ideal to quantify TSPO PET image in the brain. Therefore, the use of classical compartmental modeling or endothelial modeling of TSPO PET imaging [64, 65] needs to be considered to quantify [^18^F]BS224 PET in a more generalized setting before clinical study. Moreover, it will be of more interest to evaluate a subsequent comparative study of [^18^F]BS224 with reported TSPO ligands in preclinical or clinical study to prove beyond a reasonable doubt to be a next-generation TSPO ligand.

The results in LPS-induced inflammation and ischemic stroke rat models suggested that [^18^F]BS224 could display high signal-to-background ratio in brain imaging because of the low effect of blood pools during early imaging and the absence of skull uptake due to the high in vivo stability of aromatic fluorine. Taken as a whole, these data can represent a valuable starting point for designing the next generation of TSPO ligands.

## Conclusion

We successfully synthesized a new TSPO PET ligand, [^18^F]BS224, using two different precursors. In aromatic ^18^F-fluorination of [^18^F]BS224, boronic acid pinacol ester, precursor **6**, showed higher RCY and required relatively milder reaction conditions than iodotoluene tosylate, precursor **7**. Results of docking simulations offered a molecular rationale for the low sensitivity to the rs6971 polymorphism exhibited by this new TSPO PET ligand. The LAB/HAB IC_50_ ratio indicated that the binding affinity of BS224 is less affected by LAB and is comparable to that of the well-known PK 11195. Our PET imaging results suggest that [^18^F]BS224 displays a high target to background ratio during early imaging of lesioned brain regions with abnormal TSPO expression and provides a clearly visible image of the inflammatory lesions in both animal models without skull uptake. Based on these findings, we do believe that [^18^F]BS224 might be a promising next-generation TSPO PET ligand to gauge neuroinflammatory disease-relevant areas in a broad range of patients irrespective of the common rs6971 polymorphism.

## Supplementary Information

Below is the link to the electronic supplementary material.

Supplementary file1 (DOCX 4279 kb)
