# Supplementary material for: Radiosynthesis and characterization of [18F]BS224: a next-generation TSPO PET ligand insensitive to the rs6971 polymorphism
Source: Eur J Nucl Med Mol Imaging. 2021 Nov 16;49(1):110–24. doi: 10.1007/s00259-021-05617-4 (PMC8712300; doi:10.1007/s00259-021-05617-4)
Supplement: Supplementary file 1 — Supplementary file1 (DOCX 4279 kb) [file 259_2021_5617_MOESM1_ESM.docx]

**Supplementary Information**

**Radiosynthesis and Characterization of [^18^F]BS224: A Next-Generation TSPO PET Ligand Insensitive to the rs6971 Polymorphism**

Sang Hee Lee^1,2,†^, Nunzio Denora^3,†^, Valentino Laquintana^3^, Giuseppe Felice Mangiatordi^4^, Angela Lopedota^3^, Antonio Lopalco^3^, Annalisa Cutrignelli^3^, Massimo Franco^3^, Pietro Delre^4,5^, In Ho Song^1^, Hye Won Kim^1,2,^ Su Bin Kim^1,2^, Hyun Soo Park^1^, Kyungmin Kim^6,7,8^, Seok-Yong Lee^6,7,8^, Hyewon Youn^6,8*^, Byung Chul Lee^1,9*^, Sang Eun Kim^1,9,10*^

*^1^Department of Nuclear Medicine, Seoul National University Bundang Hospital, Seoul National University College of Medicine, Seongnam 13620, Republic of Korea*

*^2^Department of Transdisciplinary Studies, Graduate School of Convergence Science and Technology, Seoul National University, Seoul 08826, Republic of Korea*

*^3^Department of Pharmacy – Drug Sciences, University of Bari “A. Moro”, 70121 Bari, Italy*

*^4^Institute of Crystallography, National Research Council, Via G. Amendola 122/O, 70126 Bari, Italy*

*^5^Department of Chemistry, University of Bari “A. Moro”, Via E. Orabona, 4, I-70125 Bari, Italy*

*^6^Department of Nuclear Medicine, Seoul National University Hospital, Seoul 03080, Republic of Korea*

*^7^Department of Biomedical Sciences, Seoul National University Graduate School, Seoul 03080, Republic of Korea*

*^8^Laboratory of Molecular Imaging and Therapy, Cancer Research Institute, Seoul National University College of Medicine, Seoul 03080, Republic of Korea*

*^9^Center for Nanomolecular Imaging and Innovative Drug Development, Advanced Institutes of Convergence Technology, Suwon 16229, Republic of Korea*

*^10^Department of Molecular Medicine and Biopharmaceutical Sciences, Graduate School of Convergence Science and Technology, Seoul National University, Seoul 08826, Republic of Korea*

**Corresponding authors:**

Sang Eun Kim, M.D., Ph.D., [kse@snu.ac.kr](mailto:kse@snu.ac.kr) P: +82-31-787-7671, Fax: +82-31-787-4018; Byung Chul Lee, Ph.D., [leebc@snu.ac.kr](mailto:leebc@snu.ac.kr) P: +82-31-787-2956, Fax: +82-31-787-4072; Hyewon Youn, Ph.D., hwyoun@snu.ac.kr P: +82-2-3668-7026, Fax: +82-2-745-7690

**Contents**

*Experimental Section* S3

*Supplementary Figures* S9

**Fig. S1** NMR Spectra (^1^H and ^13^C NMR) of Compounds (**1**-**7**) S10

**Fig. S2** Radio-TLC Profiles for ^18^F-labeling of [^18^F]BS224 S19

**Fig. S3** HPLC Spectra of the Crude Mixture of [^18^F]BS224 After Aromatic ^18^F-Fluorination S20

**Fig. S4** HPLC Spectra of Co-injection with [^18^F]BS224 and BS224 S21

**Fig. S5** Radio-TLC Profiles of [^18^F]BS224 in Human Serum S22

**Fig. S6** Results of Binding (a) and Competitive Inhibition Assay (b) of BS224 S23

**Fig. S7** Regional Time-acivity Curves of [^18^F]BS224 in Normal Mice S24

**Fig. S8** HPLC Profiles of [^18^F]BS224 for In Vivo Radiometabolic Stability S25

**Fig. S9** Autoradiography Images of [^18^F]BS224 Binding in MCAO Rat Brain Slices S29

*Supplementary Table*

**TABLE S1.** Log D Measurement of [^18^F]BS224 S30

**TABLE S2.** Measurement of Plasma Free Fraction for [^18^F]BS224 S31

**TABLE S3.** Estimated Human Absorbed Doses and Effective of [^18^F]BS224 from Normal Mice Data S32

*References* S33

**Experimental Section**

Commercial reagent grade chemicals were used without further purification unless otherwise specified. Flash column chromatography was performed with silica gel (Merck, 230-400 mesh, ASTM). All reaction was monitored by pre-coated plates (Merck, silica gel 60F254). Elemental analyses were carried out with a Eurovector model Euro EA 3000 elemental analyzer (Eurovector Srl). Mass spectra were recorded on a Hewlett-Packard 5995c GC-MS low-resolution spectrometer (Agilent) and electrospray mass spectrometry (ESI-MS) were performed on an Agilent 1100 LC-MSD trap system instrument. IR spectra were recorded using a Perkin-Elemer 1725 series FTIR spectrophotometer (Perkin-Elmer Corporation) and samples were prepared using KBr pellets. Melting points were determined in open capillary tubes on a Büchi apparatus. ^1^H and ^13^C NMR spectra were obtained by Varian 400-MR (Agilent) spectrometer at ambient temperature. Chemical shifts were reported in parts per million (ppm, δ units). In radiochemistry, H_2_^18^O was purchased from Taiyo Nippon Sanso Corporation. Fluorine-18 was produced by ^18^O(p,n)^18^F reaction through proton irradiation using a cyclotron. HPLC purification was performed with a Gilson 322. Radio TLC was analyzed on a AR-2000 radio-TLC imaging scanner (Bioscan Inc.). In vitro stability experiment was carried out at 37 °C using a block heater (Digi-Block Laboratory Device Inc.). All radioactivities were measured using Dose Calibrator Model VDC-505 (Comecer). PET/CT imaging of rats was performed by nanoPET/CT (Mediso). [^3^H]PK11195 was purchased from Perkin-Elmer. Autoradiography of [^18^F]BS224 in the brain slices was conducted by a GE FLA-7000 Phosphor Imaging Typhoon scanner (GE Healthcare).

**General Procedure for Compound 2a and 2b**

A mixture of 3-(4-fluorobenzoyl)propionic acid (5.0 g, 25.5 mmol) and *N*,*N*’-dipropylamine 1,1’-carbonyldimidazole (CDI, 4.5 g, 28.0 mmol) in anhydrous tetrahydrofuran (THF, 50 mL) was stirred at room temperature for 30 min. After adding of *N*,*N*’-dipropylamine (4.2 mL, 30.6 mmol) and triethylamine (Et_3_N, 4.6 mL, 33.1 mmol), the reaction mixture were stirred at the same temperature for 4 h. Solvent was removed under reduced pressure and the mixture was diluted with ethyl acetate (30 mL) washed with 0.1 N HCl (20 mL) with two times, and the extracted organic layer was dried over anhydrous Na_2_SO_4_ and evaporated under reduced pressure. The crude product was purified by silica gel column chromatography using 30% ethyl acetate-hexane as the eluent, to afford 4-(4-fluorophenyl)-4-oxo-*N*,*N*-dipropylbutanamide **2a** as a white solid (5.7 g, 80%). M.p., 175-177 °C; ^1^H NMR (400 MHz, CDCl_3_) δ 0.86 (t, *J* = 7.4 Hz, 3H), 0.95 (t, *J* = 7.4 Hz, 3H), 1.53 (q, *J* = 7.4 Hz, 2H), 1.65 (q, *J* = 7.4 Hz, 2H), 2.77 (t, *J* = 7.4 Hz, 2H), 3.2-3.4 (m, 6H), 7.12 (d, *J* = 6.8 Hz, 2H), 7.90 (d, *J* = 6.8 Hz, 2H); ^13^C NMR (100 MHz, CDCl_3_) δ 198.0, 171.2, 167.1, 164.6, 133.6, 133.5, 130.9, 130.8, 115.8, 115.6, 49.7, 47.9, 33.8, 27.3, 22.3, 21.1, 11.5, 11.4; MS (ESI) *m/z* (% relative to the base peak) 279 (5%, M+), 179 (base); IR (KBr cell cm^-1^), ν = 1640 (C=O), 1685 (C=O, CONPr_2_); Elemental Analysis, calcd for C_16_H_22_FNO_2_: C, 68.79; H, 7.94; N, 5.01 %, found: C, 68.94; H, 7.90; N, 5.03 %.

**4-(4-Bromophenyl)-4-oxo-*N*,*N*-dipropylbutanamide** (**2b**).

The compound **2b** was prepared following the same procedure shown above and starting from 3-(4-bromobenzoyl)propionic acid (6.5 g, 25.5 mmol). Yellow solid (76%); M.p., 210-213 °C; ^1^H NMR (400 MHz, CDCl_3_) δ 0.86 (t, *J* = 7.4 Hz, 3H), 0.95 (t, *J* = 7.4 Hz, 3H), 1.53 (q, *J* = 7.4 Hz, 2H), 1.65 (q, *J* = 7.4 Hz, 2H), 2.77 (t, *J* = 7.4 Hz, 2H), 3.2-3.4 (m, 6H), 7.58 (d, *J* = 6.8 Hz, 2H), 7.86 (d, *J* = 6.8 Hz, 2H); ^13^C NMR (100 MHz, CDCl_3_) δ 198.6, 171.1, 135.8, 132.0, 131.9, 129.8, 129.7, 128.2, 49.7, 47.9, 33.9, 27.3, 22.3, 21.1, 11.6, 11.5, 11.4; MS (ESI) *m/z* (% relative to the base peak) 339 (5%, M^+^), 239 (base); IR (KBr cell cm^-1^), ν = 1639 (C=O), 1687 (C=O, CONPr_2_); Elemental Analysis, calcd for C_16_H_22_BrNO_2_: C, 56.48; H, 6.52; N, 4.12 %, found: C, 56.59; H, 6.50; N, 4.11 %

**General Procedure for Compound 3a and 3b**

To a stirred solution of **2a** (3.0 g, 10.7 mmol) in CCl_4_ (50 mL) was dropwise added a solution of Br_2_ (0.60 mL, 11.8 mmol) in CCl_4_ (1 mL) at room temperature. After stirring for 2 h, the solvent was removed under reduced pressure and the mixture was diluted with a saturated solution of NaHCO_3_ (50 mL) and extracted with ethyl acetate (3 × 50 mL). The organic layer was removed, dried over anhydrous sodium sulfate and taken to dryness under reduced pressure. The crude product was purified by silica gel column chromatography using 20% ethyl acetate-hexane as the eluent, to afford 3-bromo-4-(4-fluorophenyl)-4-oxo-*N*,*N*-dipropylbutanamide (**3a**) as a white solid (3.3 g, 87%). M.p.,191-193 °C; ^1^H NMR (400 MHz, CDCl_3_) δ 0.83 (t, *J* = 7.4 Hz, 3H), 0.98 (t, *J* = 7.4 Hz, 3H), 1.49 (q, *J* = 7.4 Hz, 2H), 1.66 (q, *J* = 7.4 Hz, 2H), 3.02 (dd, *J*_1_ = 16.0 Hz, *J*_2_ = 4.4 Hz, 1H), 3.1-3.4 (m, 4H), 3.57 (dd, *J*_1_ = 16.0 Hz, *J*_3_ = 9.9 Hz, 1H), 5.59 (dd, *J*_3_ = 9.9 Hz, *J*_2_ = 4.4 Hz, 1H), 7.12 (d, *J* = 6.8 Hz, 2H), 7.90 (d, *J* = 6.8 Hz, 2H); ^13^C NMR (100 MHz, CDCl_3_) δ 191.8, 169.3, 167.4, 164.9, 131.9, 131.8, 130.8, 130.7, 116.2, 115.9, 49.7, 47.6, 40.7, 38.5, 22.2, 21.0, 11.4; MS (ESI) *m/z* (% relative to the base peak) 358 (0.9%, M^+^), 259 (base); IR (KBr cell cm^-1^), ν = 1690 (C=O, COCHBr), 1687 (C=O, CONPr_2_); Elemental Analysis, calcd for C_16_H_21_BrFNO_2_: C, 53.64; H, 5.91; N, 3.91 %, found: C, 53.73; H, 5.89; N, 3.93 %

**3-Bromo-4-(4-bromophenyl)-4-oxo-*N*,*N*-dipropylbutanamide (3b).**

Orange solid (90%); M.p., 220-223 °C; ^1^H NMR (400 MHz, CDCl_3_) δ 0.83 (t, *J* = 7.4 Hz, 3H), 0.98 (t, *J* = 7.4 Hz, 3H), 1.49 (q, *J* = 7.4 Hz, 2H), 1.66 (q, *J* = 7.4 Hz, 2H), 3.01 (dd, *J*_1_ = 16.0 Hz, *J*_2_ = 4.4 Hz, 1H), 3.1-3.4 (m, 4H), 3.57 (dd, *J*_1_ = 16.0 Hz, *J*_3_ = 9.9 Hz, 1H), 5.59 (dd, *J*_3_ = 9.9 Hz, *J*_2_ = 4.4 Hz, 1H), 7.62 (d, *J* = 6.8 Hz, 2H), 7.91 (d, *J* = 6.8 Hz, 2H); ^13^C NMR (100 MHz, CDCl_3_) δ 192.4, 169.3, 133.2, 132.3, 132.1, 130.7, 130.6, 130.5, 128.9, 49.7, 47.6, 40.6, 38.5, 38.4, 22.2, 21.0, 11.5, 11.4; MS (ESI) *m/z* (% relative to the base peak) 419 (0.5%, M^+^), 319 (base); IR (KBr cell cm^-1^), ν = 1689 (C=O, COCHBr), 1683 (C=O, CONPr_2_); Elemental Analysis, calcd for C_16_H_21_Br_2_NO_2_: C, 45.85; H, 5.05; N, 3.34 %, found: C, 46.03; H, 5.03; N, 3.35 %

**General Procedure for Compound 1 and 4**

To a solution of compound **3a** (2.0 g, 5.6 mmol) in anhydrous *N*,*N*-dimethylformamide (DMF, 25 mL) was added 2-amino-3,5-dichloropyridine (1.2 g, 7.3 mmol) and heated at 120 ^o^C for 18 h. The solvent was evaporated under reduced pressure and the solid residue was dissolved in ethyl acetate (20 mL) and washed with 0.1 N HCl (30 mL) for three times. The organic layer was dried over anhydrous Na_2_SO_4_, then filtered and evaporated. The crude product was purified by silica gel column chromatography using 20% ethyl acetate-hexane as the eluent, to give 2-(-2-(4-fluorophenyl)-6,8-dichloro-imidazo[1,2-a]pyridin-3-yl)-*N*,*N*-dipropylacetamide **1** (BS224) as a white solid (1.0 g, 45%). M.p., 123-125 °C; ^1^H NMR (400 MHz, CDCl_3_) δ 0.77 (t, *J* = 7.4 Hz, 3H), 0.86 (t, *J* = 7.4 Hz, 3H), 1.2-1.3 (m, 4H), 3.12 (t, *J* = 7.7 Hz, 2H), 3.30 (t, *J* = 7.7 Hz, 2H), 4.04 (s, 2H), 7.30 (d, *J* = 1.9 Hz, 1H), 7.23 (d, *J* = 8.2 Hz, 2H), 7.56 (d, *J* = 8.2 Hz, 2H), 8.23 (d, *J* = 1.9 Hz, 1H); ^13^C NMR (100 MHz, CDCl_3_) δ 167.2, 164.3, 161.8, 144.7, 141.2, 130.9, 130.8, 130.7, 130.6, 130.0, 129.9, 124.9, 124.7, 123.4, 121.8, 121.7, 120.0, 117.3, 116.1, 115.9, 115.8, 115.7, 50.0, 48.2, 30.3, 22.4, 21.0, 11.5, 11.4, 11.2, 11.1; MS (ESI) *m/z* 421 (M-H)^+^; IR (KBr cell cm^-1^), ν = 1640 (C=O, CONPr_2_); Elemental Analysis, calcd for C_21_H_22_Cl_2_FN_3_O: C, 59.72; H, 5.25; N, 9.95 %, found: C, 59.77; H, 5.21; N, 9.98 %.

**2-(2-(4-Bromophenyl)-6,8-dichloro-imidazo[1,2-a]pyridin-3-yl)-*N*,*N*-dipropylacetamide (4).**

White solid (55%); M. P., 195-197 °C; ^1^H NMR (400 MHz, CDCl_3_) δ 0.77 (t, *J* = 7.4 Hz, 3H), 0.86 (t, *J* = 7.4 Hz, 3H), 1.2-1.3 (m, 4H), 3.12 (t, *J* = 7.7 Hz, 2H), 3.30 (t, *J* = 7.7 Hz, 2H), 4.04 (s, 2H), 7.30 (d, *J* = 1.9 Hz, 1H), 7.53 (d, *J* = 8.2 Hz, 2H), 7.60 (d, *J* = 8.2 Hz, 2H), 8.24 (d, *J* = 1.9 Hz, 1H);^13^C NMR (100 MHz, CDCl_3_) δ 167.4, 144.8, 141.6, 133.1, 132.3, 130.9, 125.3, 123.8, 123.1, 122.1, 120.1, 117.9, 50.4, 48.5, 30.6, 22.7, 21.4, 11.8, 11.5; MS (ESI) *m/z* 483 (M-H)^+^; IR (KBr cell cm^-1^), ν = 1698 (C=O, CONPr_2_); Elemental Analysis, calcd for C_21_H_22_BrCl_2_N_3_O: C, 52.20; H, 4.59; N, 8.70 %, found: C, 52.34; H, 4.57; N, 8.74 %.

**2-(6,8-Dichloro-2-(4-(trimethylstannyl)phenyl)imidazo[1,2-a]pyridin-3-yl)-*N*,*N*-dipropylacetamide (5).**

To a solution of hexamethylditin (0.43 mL, 2.08 mmol) and a solution of Pd(PPh_3_)_4_ (0.17 g, 0.15 mmol) in anhydrous dioxane (5 mL) were added compound **4** (0.5 g, 1.04 mmol). The reaction mixture was refluxed under stirring for 6 h. After cooling to room temperature, the reaction mixture was filtered through celite and after treating with 20 mL water was extracted using ethyl acetate (3 x 20 mL). The organic layer was removed, dried over anhydrous sodium sulfate and taken to dryness under vacuum. The crude product was purified by silica gel column chromatography using 20% ethyl acetate-hexane as the eluent, to give 2-(6,8-dichloro-2-(4-(trimethylstannyl)phenyl)imidazo[1,2-a]pyridin-3-yl)-*N*,*N*-dipropylacetamide (**5**) as a white solid (0.32 g, 54%). M.p., 125-128 °C; ^1^H NMR (400 MHz, CDCl_3_) δ 0.32 (s, 9H), 0.67 (t, *J* = 7.4 Hz, 3H), 0.86 (t, *J* = 7.4 Hz, 3H), 1.4-1.6 (m), 3.06 (t, *J* = 7.7 Hz, 2H), 3.29 (t, *J* = 7.7 Hz, 2H), 4.09 (s, 2H), 7.30 (d, *J* = 1.9 Hz, 1H), 7.59 (d, *J* = 7.8 Hz, 2H), 7.63 (d, *J* = 7.8 Hz, 2H), 8.36 (d, *J* = 1.9 Hz, 1H); ^13^C NMR (100 MHz, CDCl_3_) δ. 167.3, 145.7, 143.0, 141.3, 136.2, 133.6, 128.5, 124.7, 123.4, 122.0, 120.0, 117.4, 50.1, 48.2, 30.5, 22.4, 21.1, 11.4, 11.0, 9.4; MS (ESI) *m*/*z* 590 (M+Na)^+^. Found 590 ; IR (KBr cell cm^-1^), ν = 1640 (C=O, CONPr_2_). Elemental Analysis, calcd for C_24_H_31_Cl_2_N_3_OSn, 50.83; H, 5.51; N, 7.41 %, found: C, 51.08; H, 5.52; N, 7.43 %

**2-(6,8-Dichloro-2-(4-(4,4,5,5-tetramethyl-1,3,2-dioxaborolan-2-yl)phenyl)imidazo[1,2-a]pyridin-3-yl)-*N*,*N*-dipropylacetamide (6)**

To a solution of compound **4** (230 mg, 0.48 mmol) in DMF (15 mL), [1,1’-bis(diphenylphosphino)ferrocene]palladium(II) dichloride (41 mg, 0.05 mmol), potassium acetate (141 mg, 1.4 mmol) and bis(pinacolato)diboron (134 mg, 0.53 mmol) were added and heated at 80 °C for 6 h. After cooling, the reaction mixture was filtered through a Celite layer. The filtrate was diluted with dichoromethane (CH_2_Cl_2_, 10 mL) and then washed with water (10 mL x 3). The organic layer was dried over anhydrous Na_2_SO_4_, then filtered and concentrated under reduced pressure. The crude product was purified by silica gel column chromatography using 20% ethyl acetate-hexane as the eluent, to give 2-(6,8-dichloro-2-(4-(4,4,5,5-tetramethyl-1,3,2-dioxaborolan-2-yl)phenyl)imidazo[1,2-a]pyridin-3-yl)-*N*,*N*-dipropylacetamide (**6**) as a white solid (75.1 mg, 30%). M.p., 113-116 °C; ^1^H NMR (400 MHz, CDCl_3_) δ 0.67 (t, *J* = 7.4 Hz, 3H), 1.37 (s, 12H, CH3), 0.86 (t, *J* = 7.4 Hz, 3H, CH_3_), 1.4-1.6 (m, 4H), 3.09 (t, *J* = 7.7 Hz, 2H, CH_2_NCO), 3.29 (t, *J* = 7.7 Hz, 2H, CH_2_NCO), 4.07 (s,2H,CH_2_CO), 7.29 (d, *J* = 2.0 Hz, 1H, Ar), 7.67 (d, *J* = 8.0 Hz, 2H, Ar), 7.89 (d, *J* = 8.0 Hz, 2H, Ar), 8.28 (d, *J* = 1.9 Hz, 1H, Ar); ^13^C NMR (100 MHz, CDCl_3_) δ 167.2, 145.2, 141.2, 136.2, 135.1, 128.1, 124.6, 123.3, 121.7, 119.4, 117.6, 83.9, 49.9, 48.0, 31.6, 30.2, 24.9, 22.4, 22.2, 20.9, 14.1, 11.3, 11.0; HRMS (FAB) *m*/*z* (M+H)^+^ calcd for C_27_H_35_BCl_2_N_3_O_3_ 530.2149, found 530.2153

**(4-(6,8-Dichloro-3-(2-(dipropylamino)-2-oxoethyl)imidazo[1,2-a]pyridin-2-yl)phenyl)(p-tolyl)iodonium tosylate (7)**

To a solution of diacetoxyiodotoluene (235.2 mg, 0.7 mmol) in acetonitrile (CH_3_CN, 4 mL), p-TsOH·H_2_O (133.2 mg, 0.7 mmol) was added in an ice bath and followed by dilution with chloroform (20 mL). After stirring for 5 min at room temperature, the trimethylstannane compound **5** (260 mg, 0.46 mmol), which was prepared from compound **4**, in chloroform (4 mL) was added dropwise. The color of the reaction mixture was turned to dark green during stirring at 50 °C for 18 h. After cooling at room temperature, the organic solvent was concentrated under reduce pressure. The residue was dissolved in CH_2_Cl_2_ (10 mL) and washed with water (10 mL x 2). The organic layer was concentrated under reduced pressure to give the crude product as a dark green oil. The obtained oil was dissolved in a small volume of CH_2_Cl_2_ and diethyl ether (v/v = 1:1) and added dropwise to cold diethyl ether (10 mL) in a 15 mL centrifuge tube. After centrifuging (3400 rpm, 6 min), the collected solid was dried under vacuum to give (4-(6,8-dichloro-3-(2-(dipropylamino)-2-oxoethyl)imidazo[1,2-a]pyridin-2-yl)phenyl)(p-tolyl)iodonium tosylate (**7**) as a dark-green solid (261.1 mg, 72%). M.p., 124-131 °C; ^1^H NMR (400 MHz, CDCl_3_) δ 8.03 (d, *J* = 1.6 Hz, 1H), 7.99 (d, *J* = 8.4 Hz, 2H), 7.77 (d, *J* = 8.4 Hz, 2H), 7.64 (d, *J* = 8.4 Hz, 2H), 7.53 (bd, *J* = 7.6 Hz, 2H), 7.29 (d, *J* = 1.6 Hz, 1H), 7.11 (d, *J* = 8.0 Hz, 2H), 7.03 (bd, *J* = 7.6 Hz, 2H), 4.02 (s, 2H), 3.32-3.26 (m, 2H), 3.23-3.17 (m, 2H), 2.34 (s, 3H), 2.29 (s, 3H), 1.60-1.50 (m, 4H), 0.86 (t, *J* = 7.6 Hz, 3H), 0.80 (t, *J* = 7.2 Hz, 3H); ^13^C NMR (100 MHz, CDCl_3_) δ. 166.8, 143.3, 142.9, 141.3, 139.6, 137.4, 135.5, 135.1, 132.7, 131.8, 128.7, 126.1, 125.3, 123.7, 121.5, 120.1, 118.7, 115.0, 111.9, 50.1, 48.2, 29.8, 22.4, 21.5, 21.4, 21.1, 11.5, 11.3; HRMS (FAB) *m*/*z* (M-OTs)^+^ calcd for C_28_H_29_Cl_2_IN_3_O 620.0732, found 620.0735

**Development of the Homology Models**

The homology models of wild type rat TSPO (rTSPO) and rTSPO mutant A147T (Mut) were built based on the published solution structures of wild type (WT) mouse TSPO (mTSPO) [S1] and mTSPO Mut [S2] respectively. Notice that rat and mouse TSPO share significant sequence identity (94%) thus making us confident about the robustness of the developed homology models. Prime [S3], available from the Schrodinger suite, was used as software. More specifically, all the residues missing in the used template were constructed and refined based on the energy-based protocol and the cognate ligand of the employed templates (PK 11195) was included in the final models. The obtained homology models were pre-treated by means of the protein preparation module available from the Schrodinger suite [S4].

**Molecular Docking simulations**

BS224 was docked on the binding site of the developed homology models (rTSPO WT and rTSPO Mut). As a first step, the ligand was prepared using LigPrep [S5], available from the Schrodinger suite, in order to generate all the possible tautomers and ionization states at a pH value of 7.0 ± 2.0. Docking simulations were performed by Grid-based ligand docking with energetics (GLIDE) [S6, S7]. During the docking process, full flexibility was allowed for the ligand and the hydroxyl groups of T147 (in the A147T mutant) and T148 while the remaining part of the protein was held fixed. The extra precision (XP) protocol and the default Force Field OPLS_2005 were employed [S8]. In particular, we increased the number of poses per ligand generated in the initial phase of docking from 5000 (default setting) to 50000 and the number of poses per ligand kept for energy minimization from 400 (default setting) to 8000 in order to properly explore the conformational space of the ligands during the performed simulations. A cubic grid centered on the cognate ligand PK 11195 and having an edge of 10 Å for the inner box and 23 Å for the outer box was used. Such a protocol was tested by redocking the cognate ligand into its corresponding binding site. Satisfactory, it moved back to the original positions with a root mean square deviation (RMSD), computed taking into account all the heavy atoms, equal to 1.806 Å (TSPO WT) and 1.530 Å (TSPO Mut). Since BS224 shares a common substructure with PK 11195, docking simulations were performed restricting the explored conformational space so that only poses matching the coordinates of the common substructure (RMSD tolerance equal to 2.0 Å) were generated.

**Biodistribution and Dosimetry Study of [^18^F]BS224**

All the mice (n = 4) were anesthetized with 2% isoflurane. PET/CT images of the normal mice were obtained in the list-mode during 90 min after intravenous injection of [^18^F]BS224 (11 MBq, 0.2 mL). The CT images were used to draw region of interest (ROI) on the following organs: the brain, heart, lung, liver, stomach, urinary bladder, intestine kidneys and adrenals. To estimate the human effective dose from mouse data according to the literature [S9], decay corrected time activity curves (TACs) were obtained for each organ. The accumulated activity for each organ were calculated by using the area under curve (AUC) of TACs. The residence times in human were estimated and substituted into the IDAC-Dose 2.1 software to obtain the estimated absorbed dose for human [S10].

**LPS-induced neuroinflammatory rat model**

LPS-induced neuroinflammatory rat models were prepared as previously described [S11]. The male sprague-Dawley rats (7-8 Weeks old, Orientbio Inc.) were anesthetized with ketamine hydrochloride (50 mg/kg; Zoletil 50, Virbac, Carros, France) and xylazine hydrochloride (0.2 mg/kg; Rumpen, Bayer Korea, Seoul, Korea). The head was immobilized by stereotactic apparatus. The skull was exposed, then punctured using drill to form a small hole. Thereafter, lipopolysaccharide (LPS, 50 μg) was injected into a predetermined right brain region (AP, 0.8 mm; L, -2.7 mm; and P, -5.0 mm from the bregma) by a Hamilton syringe with a flow rate of 0.5 mL/min. LPS treatment was performed 10 minutes to prevent LPS from flowing backward in the Hamilton syringe, and then the small hole of the skull was filled with wax and the incised scalp was closed.

**Ischemic stroke rat model**

Ischemic stroke rat model was generated by middle cerebral artery occlusion (MCAO) surgery, according to the literature [S12]. Briefly, male Sprague-Dawley rats (7-8 Weeks old, Orientbio Inc.) were anesthetized with 5 % isoflurane in 30% nitrous oxide and 70% oxygen and maintained with 2 % isoflurane in 30% nitrous oxide and 70% oxygen during a MCAO surgery. The skin was incised through incision on ventral midline to expose the internal carotid artery (ICA), external carotid artery (ECA) and common carotid artery (CCA). The lower end of the CCA and both ends of the ICA were tied by thread to block blood flow. After slight incision of ICA, a nylon probe was inserted to reach the middle cerebral artery (MCA), then the blood flow was blocked for 60 minutes. Finally, the nylon probe and the threads were removed, the incised skin was sutured.

**Fig. S1** NMR Data of Compounds (**1**-**7**)

4-(4-Fluorophenyl)-4-Oxo-*N*,*N*-Dipropylbutanamide (**2a**) ^1^H NMR (400 MHz, CDCl_3_)


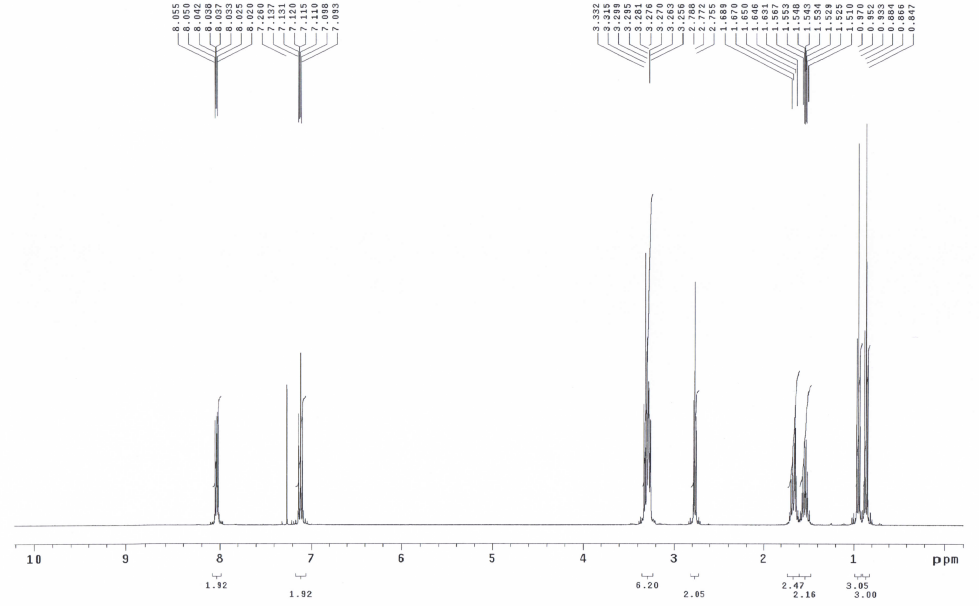


4-(4-Fluorophenyl)-4-Oxo-*N*,*N*-Dipropylbutanamide (**2a**) ^13^C NMR (100 MHz, CDCl_3_)


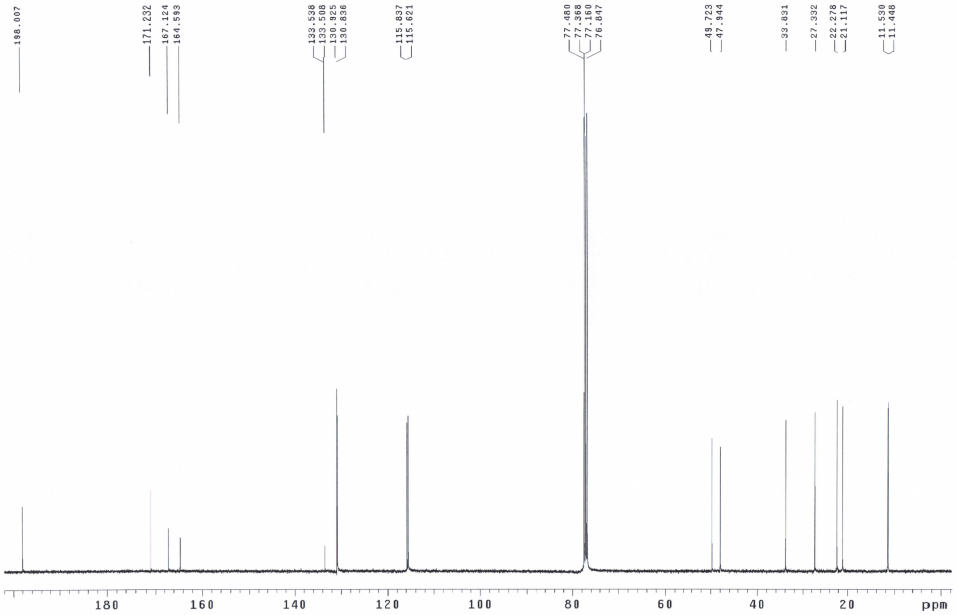


4-(4-Bromophenyl)-4-Oxo-*N*,*N*-Dipropylbutanamide (**2b**) ^1^H NMR (400 MHz, CDCl_3_)


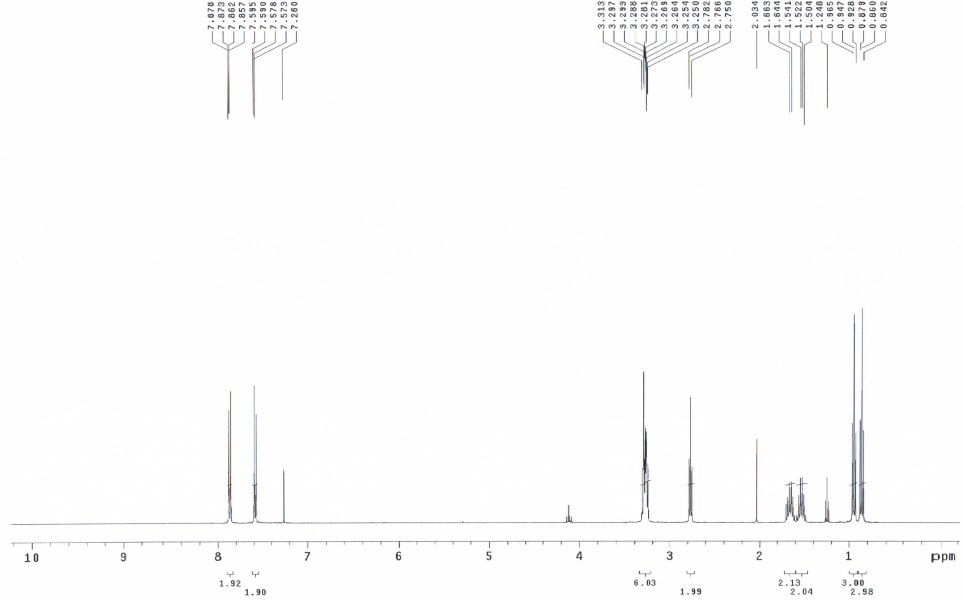


4-(4-Bromophenyl)-4-Oxo-*N*,*N*-Dipropylbutanamide (**2b**) ^13^C NMR (100 MHz, CDCl_3_)


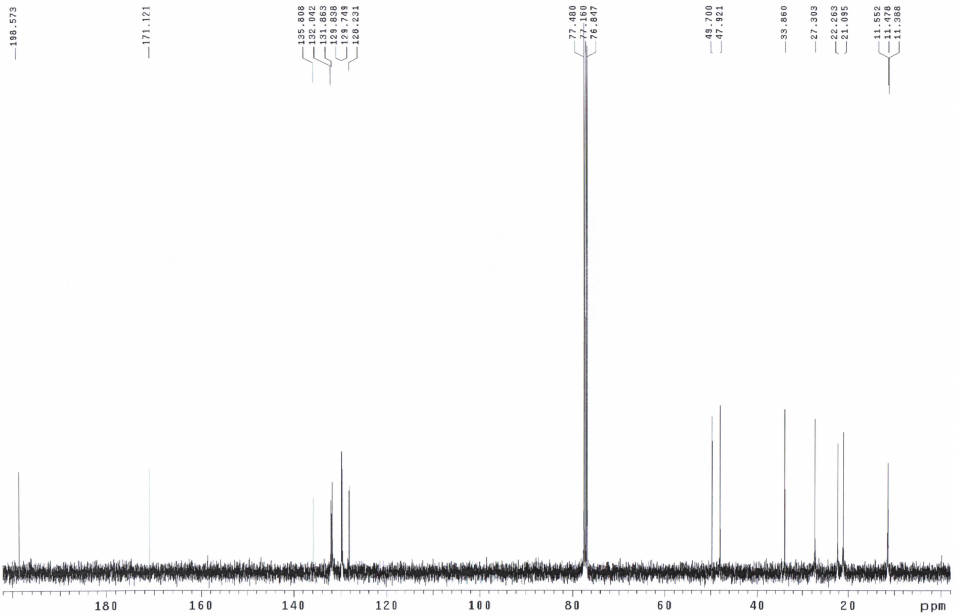


3-Bromo-4-(4-Fluorophenyl)-4-Oxo-*N*,*N*-Dipropylbutanamide (**3a**) ^1^H NMR (400 MHz, CDCl_3_)


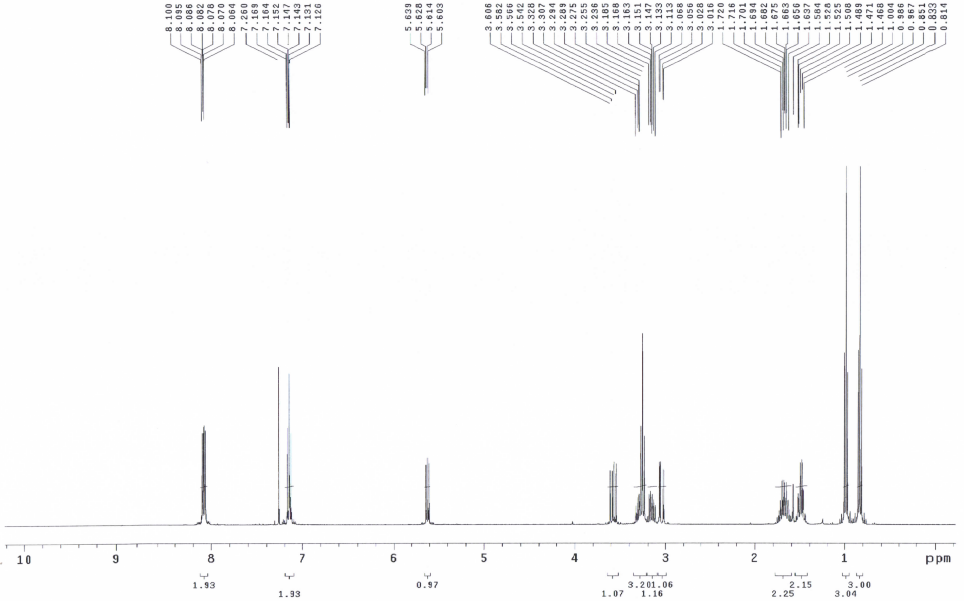


3-Bromo-4-(4-Fluorophenyl)-4-Oxo-*N*,*N*-Dipropylbutanamide (**3a**) ^13^C NMR (100 MHz, CDCl_3_)


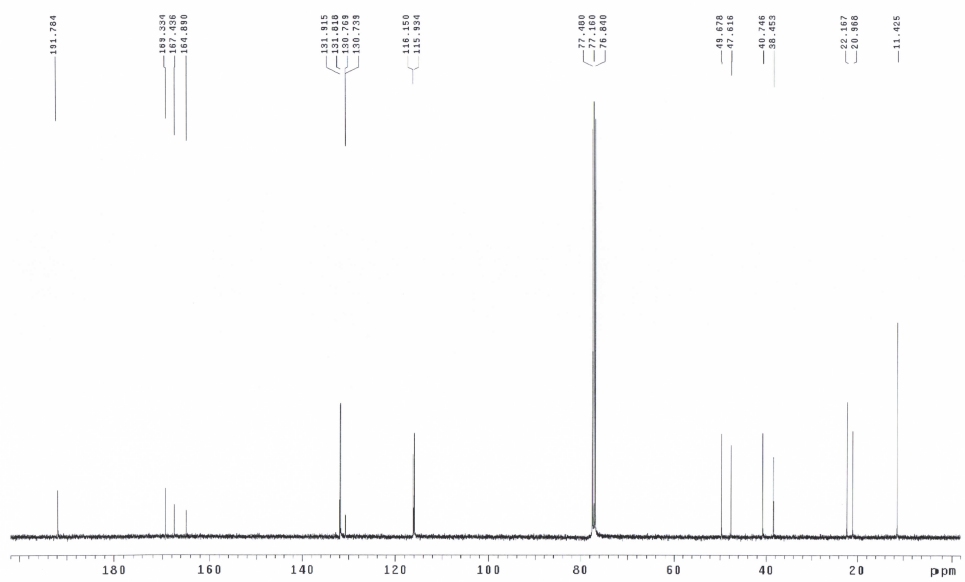


3-Bromo-4-(4-Bromophenyl)-4-Oxo-*N*,*N*-Dipropylbutanamide (**3b**) ^1^H NMR (400 MHz, CDCl_3_)


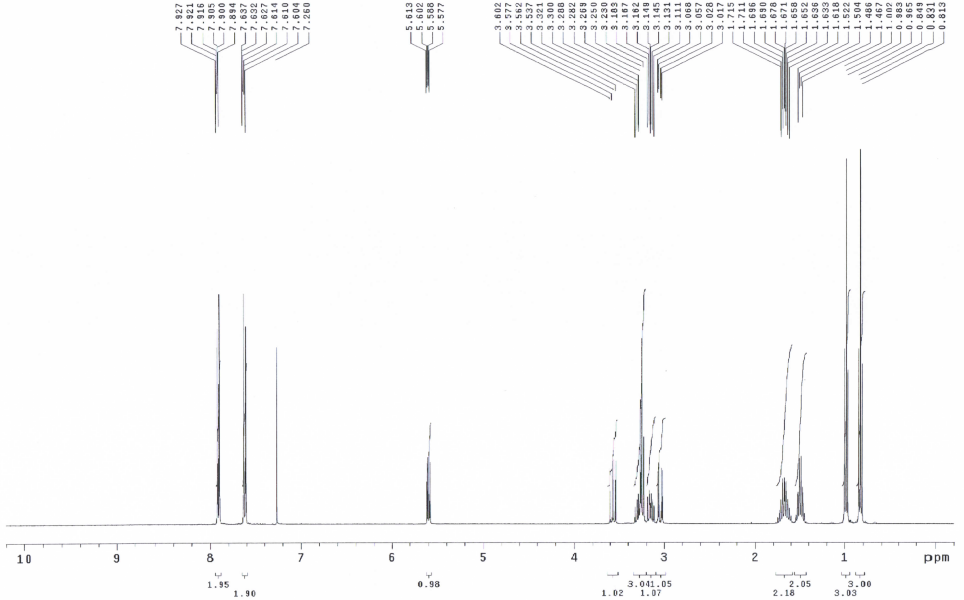


3-Bromo-4-(4-Bromophenyl)-4-Oxo-*N*,*N*-Dipropylbutanamide (**3b**) ^13^C NMR (100 MHz, CDCl_3_)


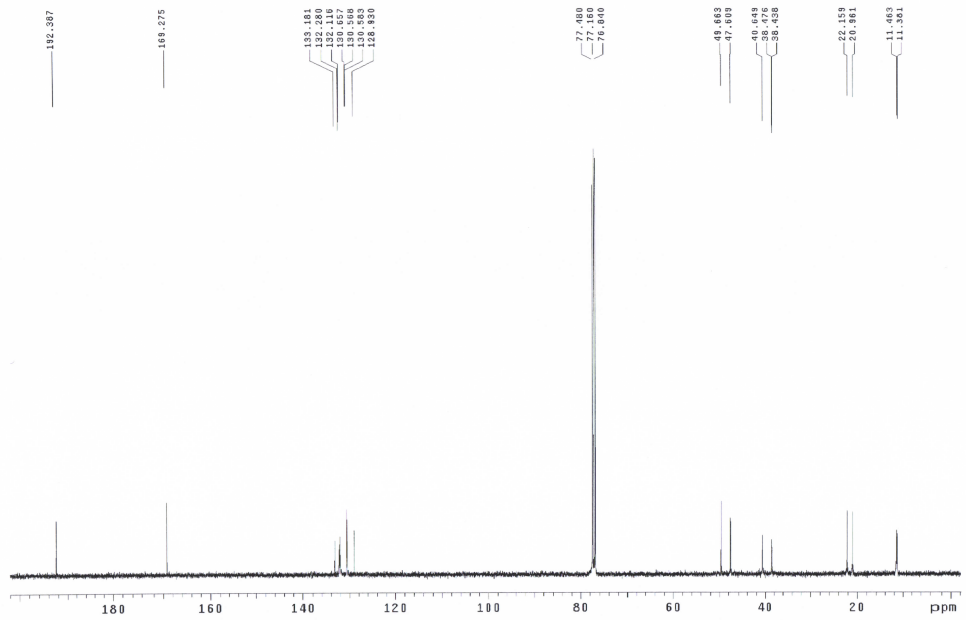


2-(2-(4-Fluorophenyl)-6,8-Dichloro-Imidazo[1,2-a]Pyridin-3-yl)-*N*,*N*-Dipropylacetamide (**1**) ^1^H NMR (400 MHz, CDCl_3_)


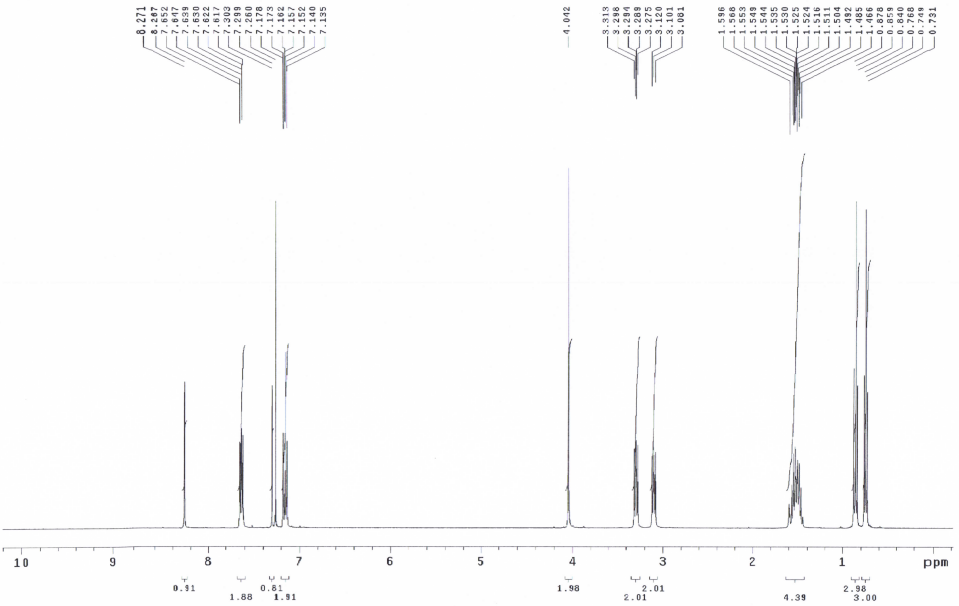


2-(2-(4-Fluorophenyl)-6,8-Dichloro-Imidazo[1,2-a]Pyridin-3-yl)-*N*,*N*-Dipropylacetamide (**1**) ^13^C NMR (100 MHz, CDCl_3_)


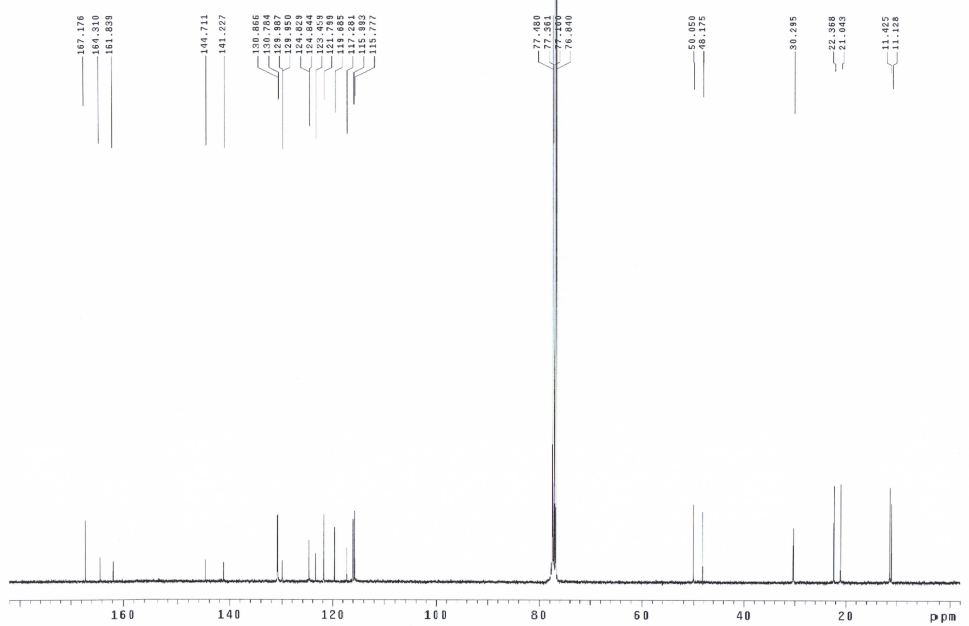


2-(2-(4-Bromophenyl)-6,8-Dichloro-Imidazo[1,2-a]Pyridin-3-yl)-*N*,*N*-Dipropylacetamide (**4**) ^1^H NMR (400 MHz, CDCl_3_)


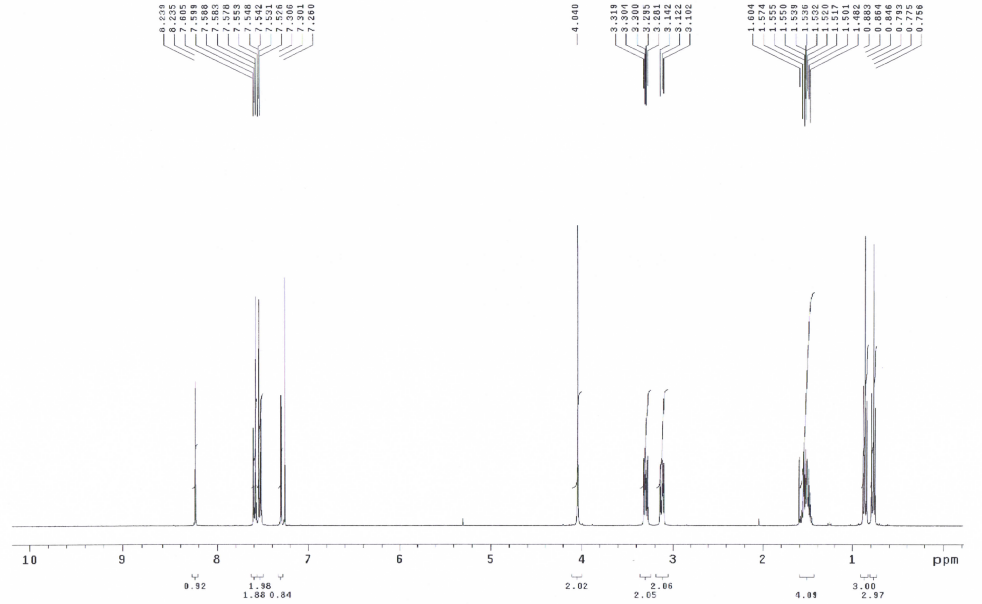


2-(2-(4-Bromophenyl)-6,8-Dichloro-Imidazo[1,2-a]Pyridin-3-yl)-*N*,*N*-Dipropylacetamide (**4**) ^13^C NMR (100 MHz, CDCl_3_)


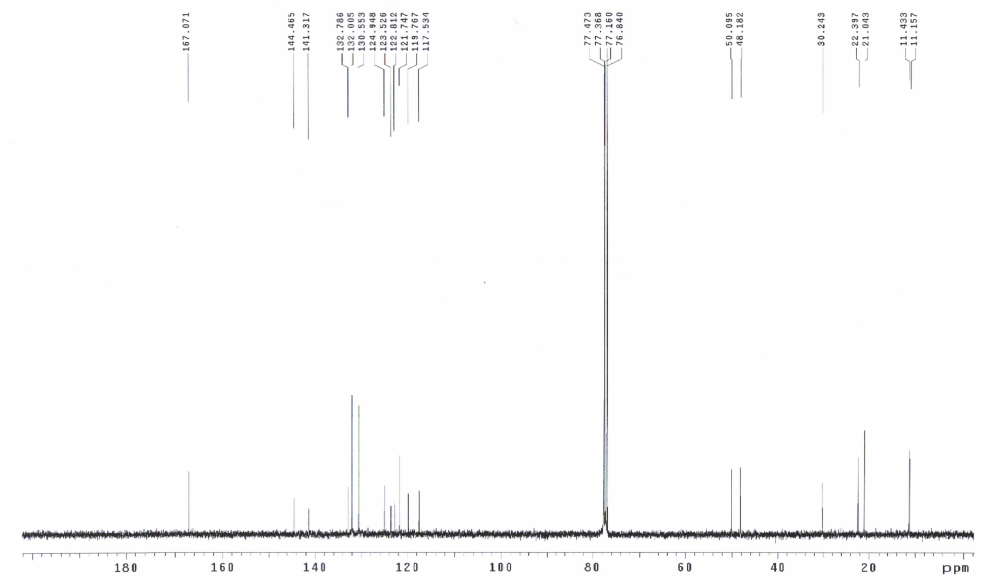


2-(2-(4-(Trimethylstannyl)Phenyl)-6,8-Dichloro-Imidazo[1,2-a]Pyridin-3-yl)-*N*,*N*-Dipropylacetamide (**5**) ^1^H NMR (400 MHz, CDCl_3_)


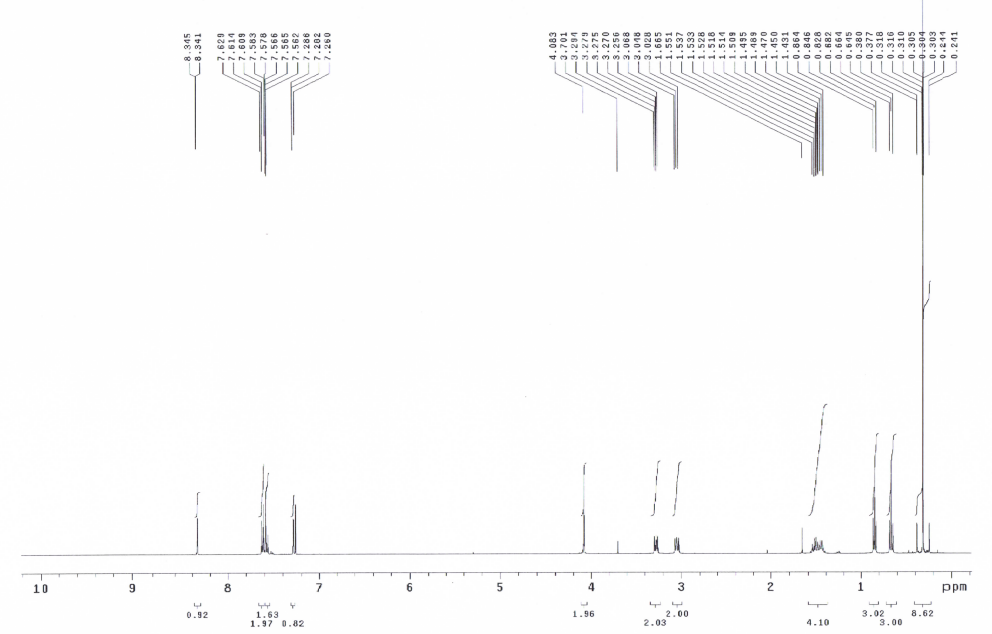


2-(2-(4-(Trimethylstannyl)Phenyl)-6,8-Dichloro-Imidazo[1,2-a]Pyridin-3-yl)-*N*,*N*-Dipropylacetamide (**5**) ^13^C NMR (100 MHz, CDCl_3_)


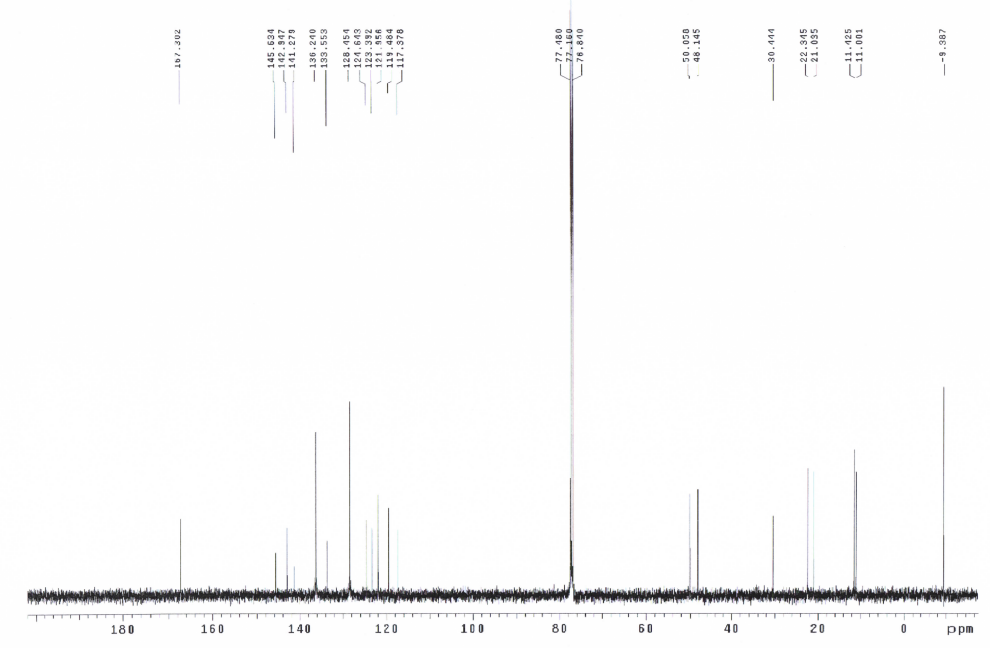


2-(6,8-Dichloro-2-(4-(4,4,5,5-Tetramethyl-1,3,2-Dioxaborolan-2-yl)Phenyl)-*N*,*N*-Dipropylacetamide (**6**) ^1^H NMR (400 MHz, CDCl_3_)


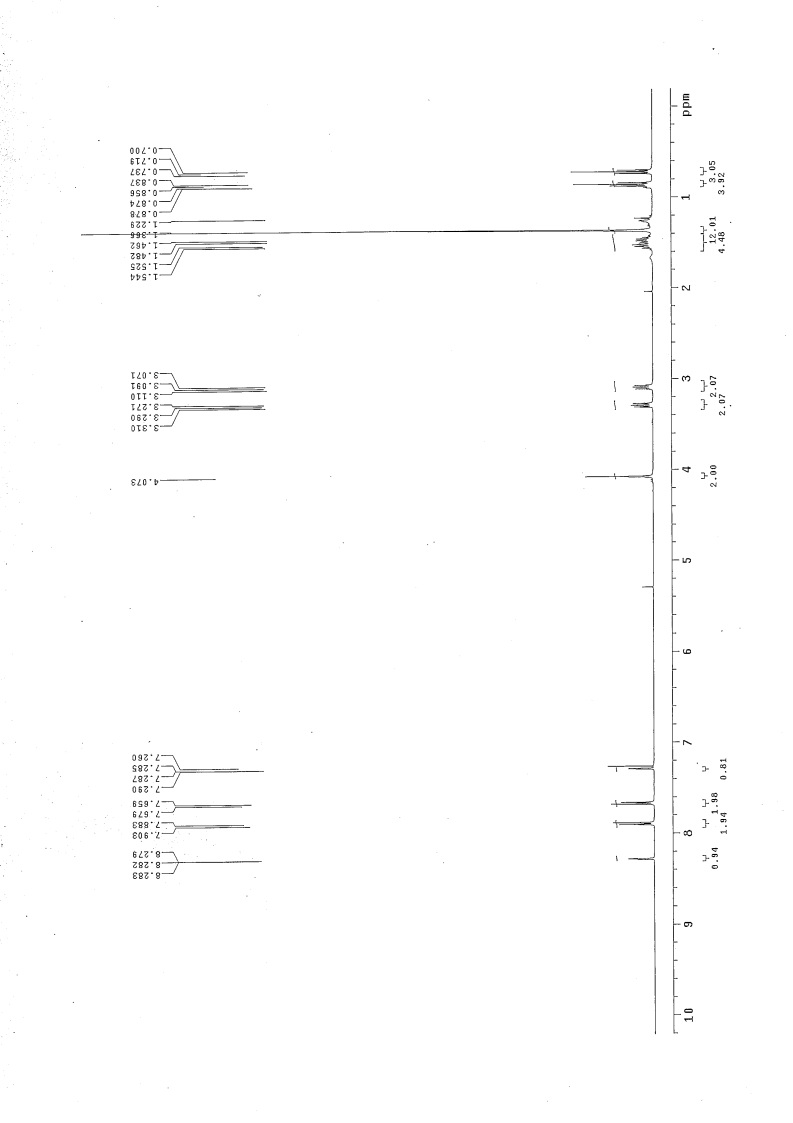


2-(6,8-Dichloro-2-(4-(4,4,5,5-Tetramethyl-1,3,2-Dioxaborolan-2-yl)Phenyl)-*N*,*N*-Dipropylacetamide (**6**) ^13^C NMR (100 MHz, CDCl_3_)


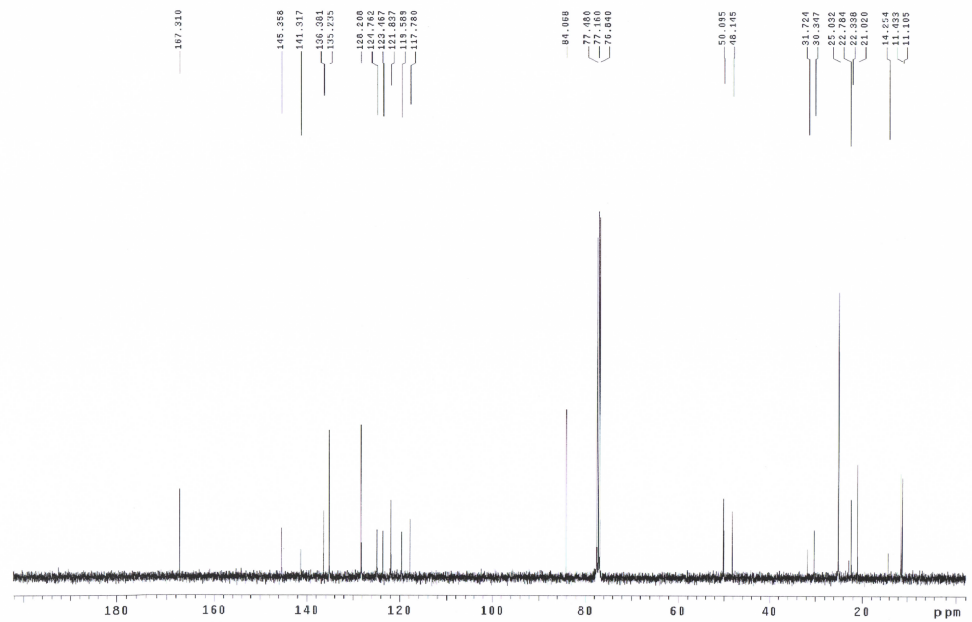


2-(2-(4-Iodotoluene Tosylate)-6,8-Dichloro-Imidazo[1,2-a]Pyridin-3-yl)-*N*,*N*-Dipropylacetamide (**7**) ^1^H NMR (400 MHz, CDCl_3_)


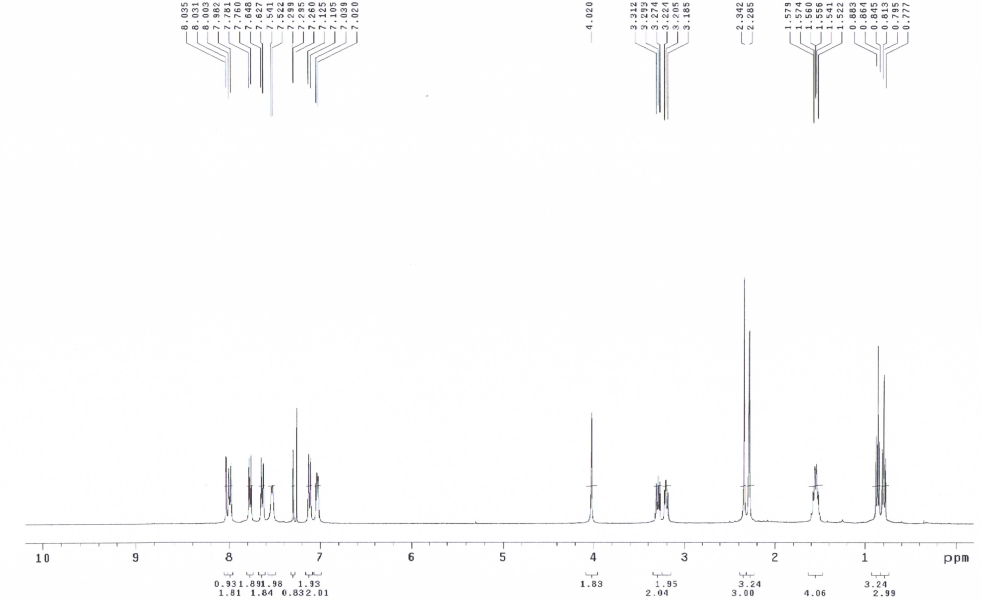


2-(2-(4-Iodotoluene Tosylate)-6,8-Dichloro-Imidazo[1,2-a]Pyridin-3-yl)-*N*,*N*-Dipropylacetamide (**7**) ^13^C NMR (100 MHz, CDCl_3_)


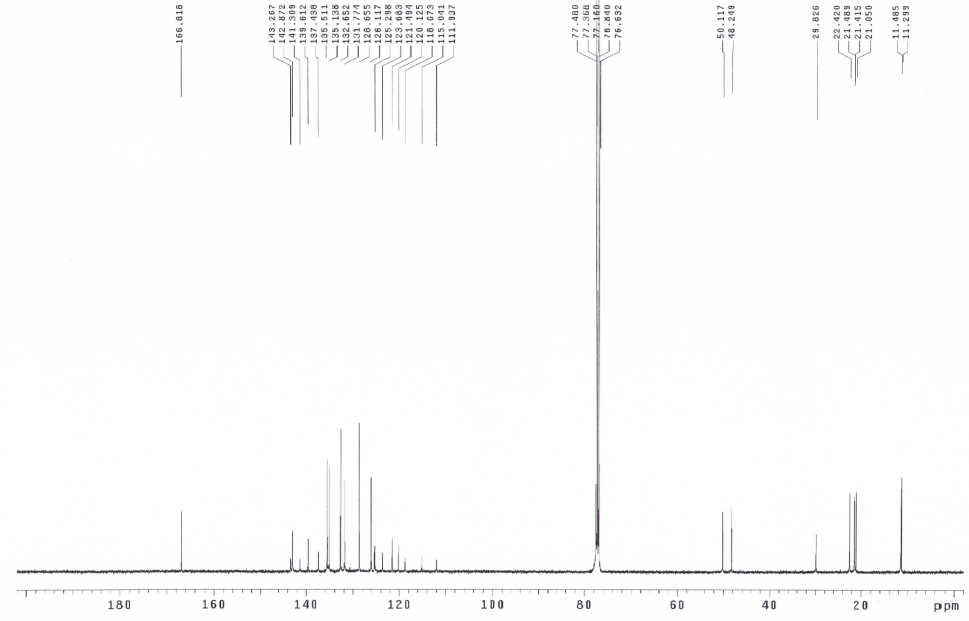


**Fig. S2** Radio-TLC Profiles for ^18^F-Labeling of [^18^F]BS224

| **A** | **B** |
| --- | --- |
| 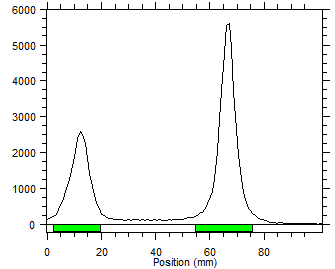 | 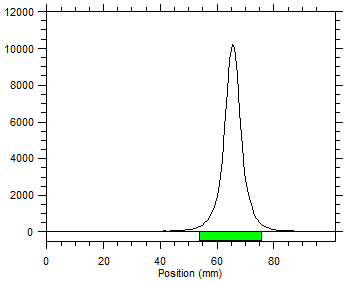 |

Radio-TLC profiles on silica gel coated glass plate. (A) The radiolabeling mixture including [^18^F]BS224; (B) The pure [^18^F]BS224 after the Sep-Pak cartridge purification.

**Radio-thin-layer-chromatography (TLC) Condition:**

System: Bioscan radio-TLC scanner (Washington, USA)

Thin-layer: Silica gel coated glass plates, 60F_254_ from Merch;10 x 100 mm (Darmstadt, Germany)

Developing eluent: 50% Ethyl acetate-Hexane (R_f_ = 0.75 for [^18^F]BS224)

% of Region of Interest of [^18^F]BS224 on the radio-TLC profile (A): 64%

**Fig. S3** HPLC Spectra of the Crude Mixture of [^18^F]BS224 After Aromatic ^18^F-Fluorination

**
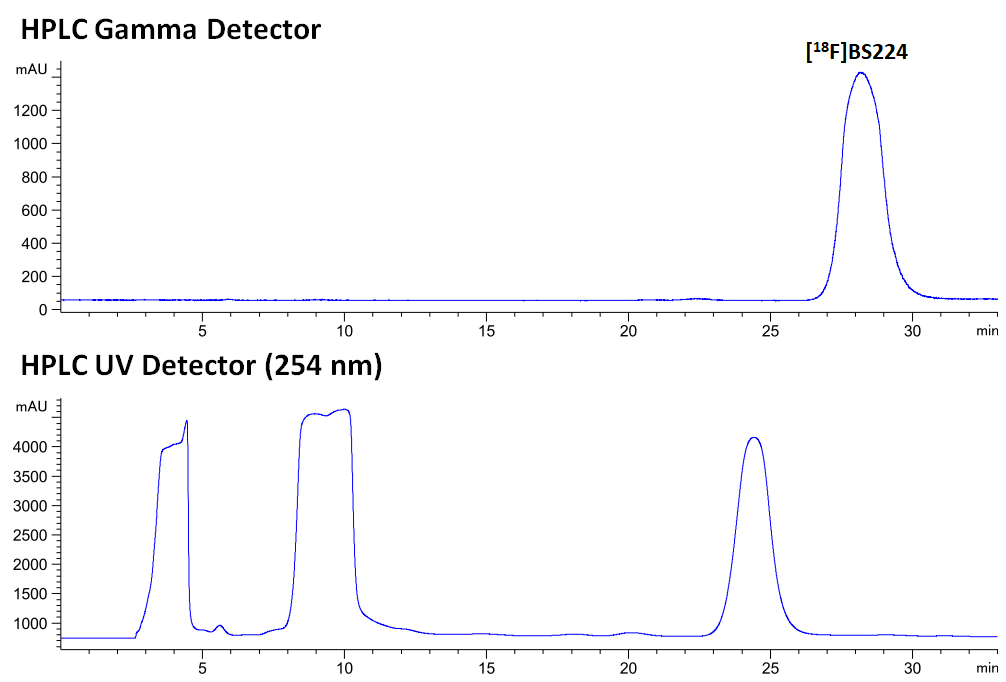
**

**HPLC Condition:**

System: Agilent Separation Products System (Santa Clara, U.S.) with UV (254 nm) and gamma-ray detectors

Column: Xterra, RP-18 C18; 4.6 x 250 mm, 10 μm

Eluent: 55% CH_3_CN/H_2_O

Flow rate: 3 mLmin^-1^

**Fig. S4** HPLC Spectra of Co-injection with [^18^F]BS224 and BS224

**
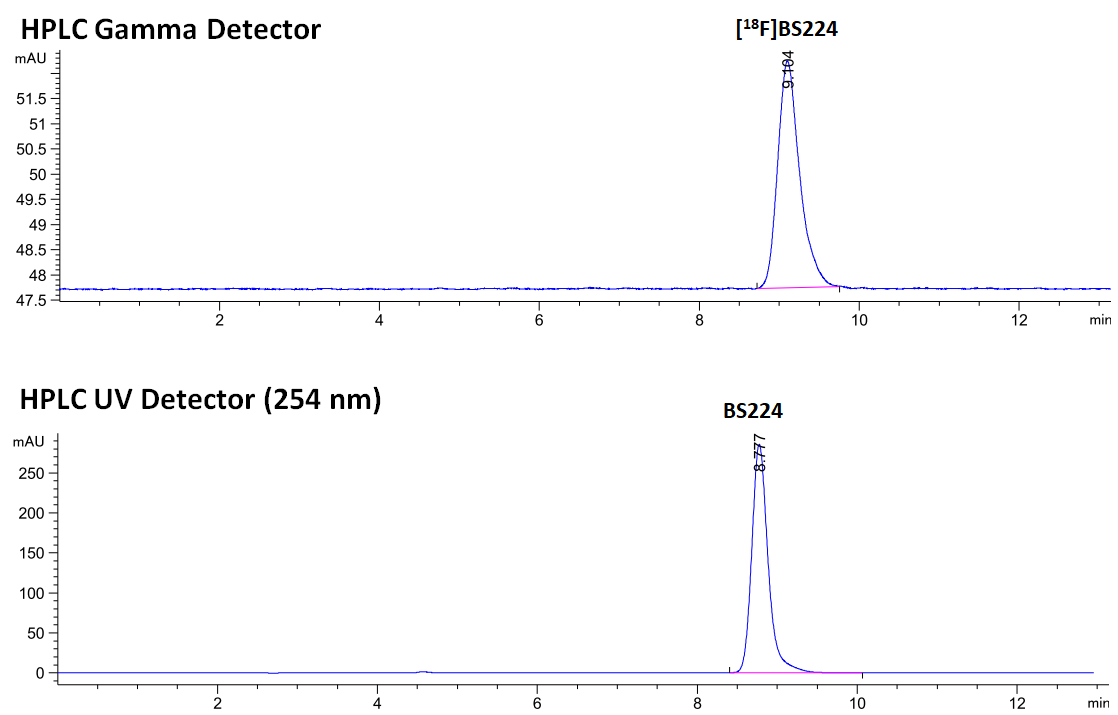
**

**HPLC Condition:**

System: Agilent Separation Products System (Santa Clara, U.S.) with UV (254 nm) and gamma-ray detectors

Analytic column: Xterra, RP-18 C18; 4.6 x 250 mm, 5μm

Eluent: 65% CH_3_CN-H_2_O

Flow rate: 1 mLmin^-1^

**Fig. S5** Radio-TLC Profiles of [^18^F]BS224 in Human Serum at 37 ^o^C

| 0 min | 15 min | 30 min | 60 min | 120 min |
| --- | --- | --- | --- | --- |
| 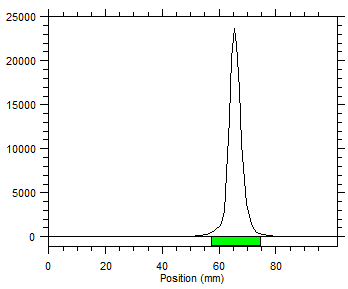 | 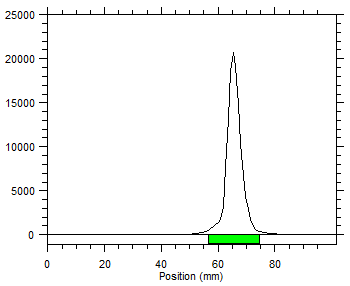 | 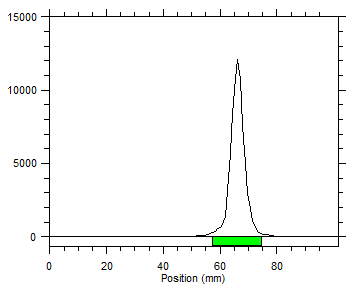 | 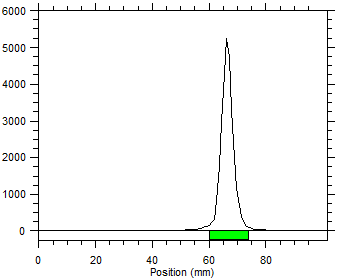 | 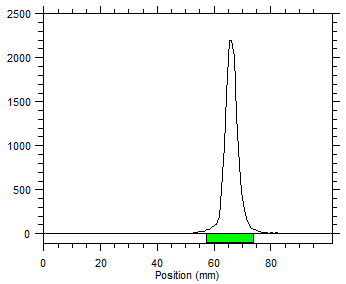 |

Radio-TLC profiles of [^18^F]BS224 in 37 ^o^C human serum at 0, 15, 30, 60 and 120 minuate.

**Radio-thin-layer-chromatography (TLC) Condition:**

System: Bioscan radio-TLC scanner (Washington, USA)

Thin-layer: Silica gel coated glass plates, 60F_254_ from Merch; 10 x 100 mm (Darmstadt, Germany)

Developing eluent: 50% Ethyl acetate-Hexane (R_f_ = 0.75 for [^18^F]BS224)

**Fig. S6** Results of Binding (**a**) and Competitive Inhibition Assay (**b**) of BS224


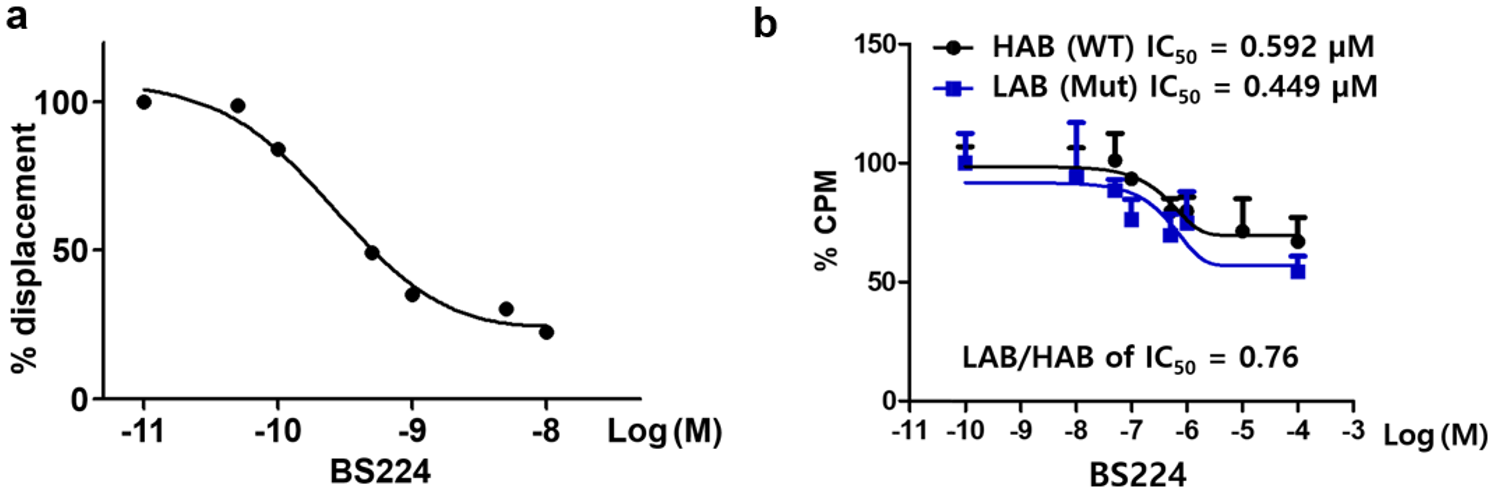


Three separate experiments performed in duplicate.

**Fig. S7** Regional Time-activity Curves of [^18^F]BS224 in Normal Mice

**
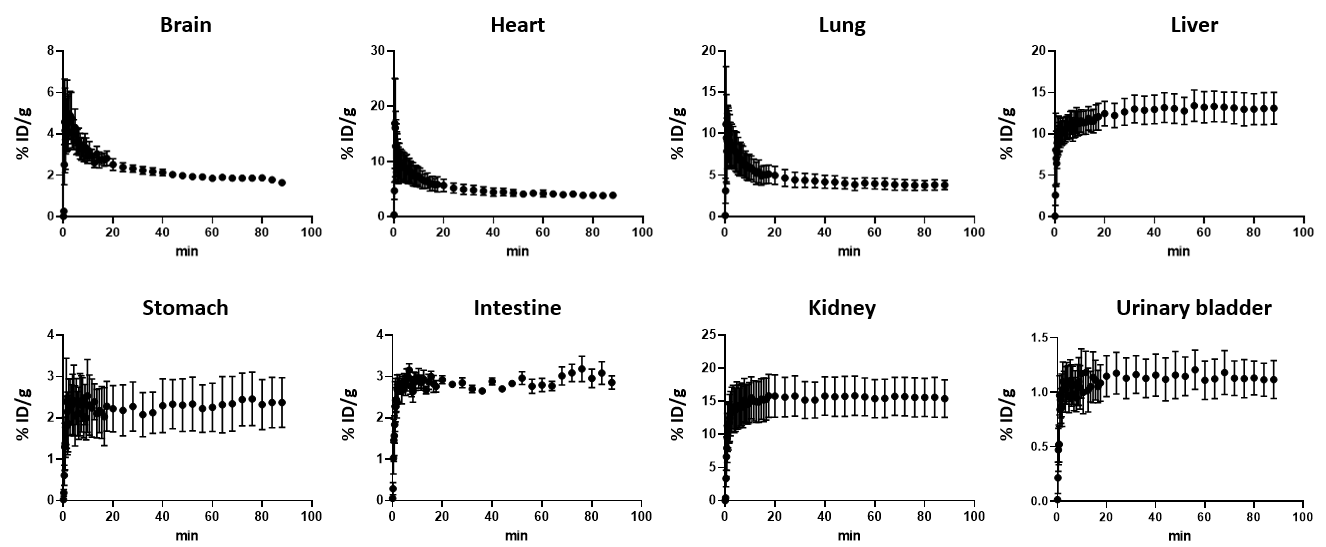
**

| **PK parameters** | **Brain** | **Liver** | **Heart** | **Lung** | **Kidney** | **Urinary Bladder** | **Intestine** | **Stomach** |
| --- | --- | --- | --- | --- | --- | --- | --- | --- |
| T_max_ [min] | 1.83±0.68 | 38.29±20.60 | 0.83±0.20 | 0.71±0.24 | 64.38±16.21 | 45.94±14.36 | 38.96±21.39 | 41.88±23.30 |
| C_max_ [%ID/g] | 5.78±1.51 | 16.21±1.95 | 20.81±8.05 | 14.38±5.88 | 16.33±3.08 | 1.35±0.22 | 3.66±0.19 | 3.12±0.81 |
| AUC [%ID/g min] | 201.75±19.25 | 1102.68±140.38 | 442.73±84.59 | 403.48±82.21 | 1345.08±260.65 | 91.44±21.23 | 250.45±7.71 | 198.98±53.18 |

Data are means ± SEM of three separate experiments.

**Fig. S8** HPLC Profiles of [^18^F]BS224 for In Vivo Radiometabolic Stability

**
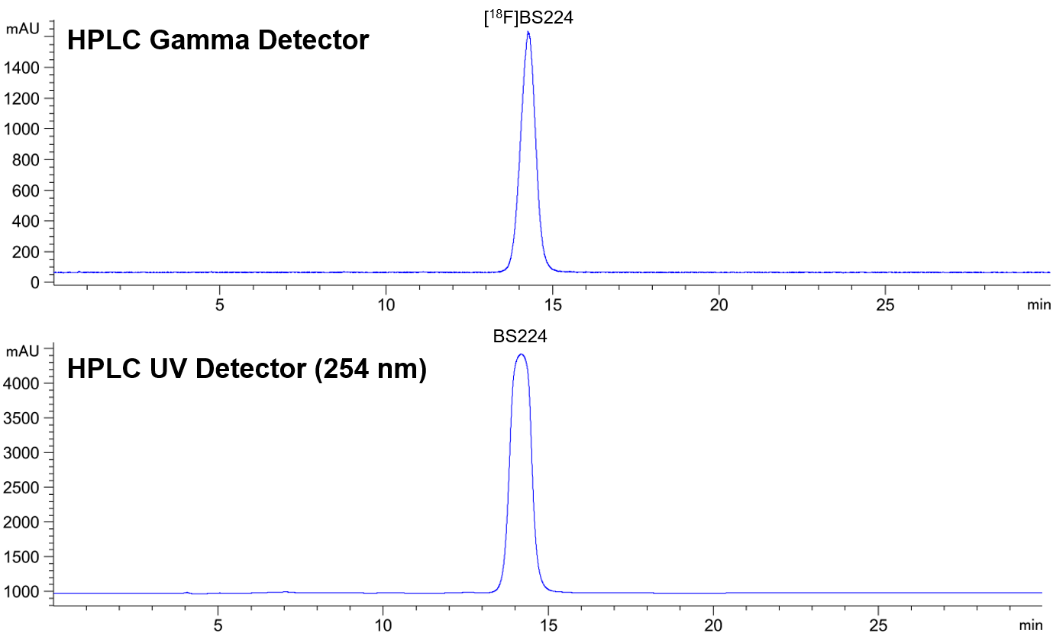
**

**HPLC Condition:**

System: Agilent Separation Products System (Santa Clara, U.S.) with UV (254 nm) and gamma-ray detectors

Column: Xterra, RP-18 C18; 4.6 x 250 mm, 10 μm

Eluent: 65% CH_3_CN/H_2_O containing 0.1% formic acid

Flow rate: 3 mLmin^-1^

**a) Brain at 30 and 90 min**

**
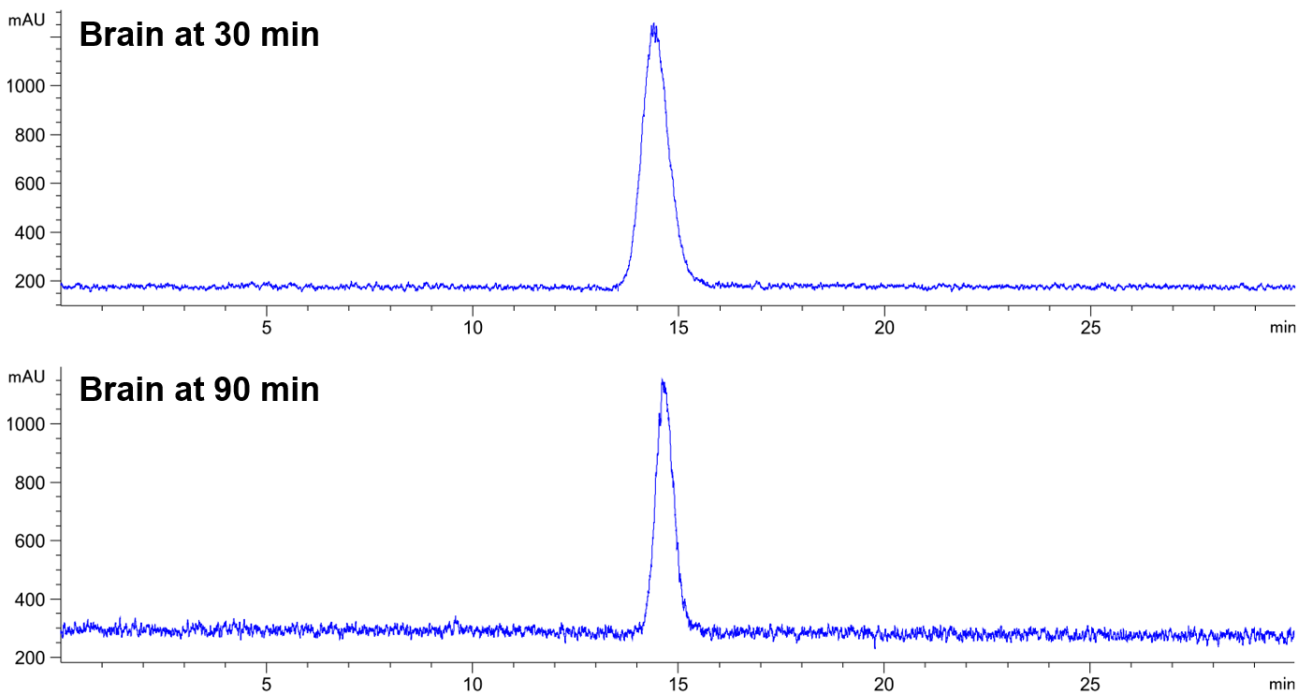
**

**b) Heart at 30 and 90 min**

**
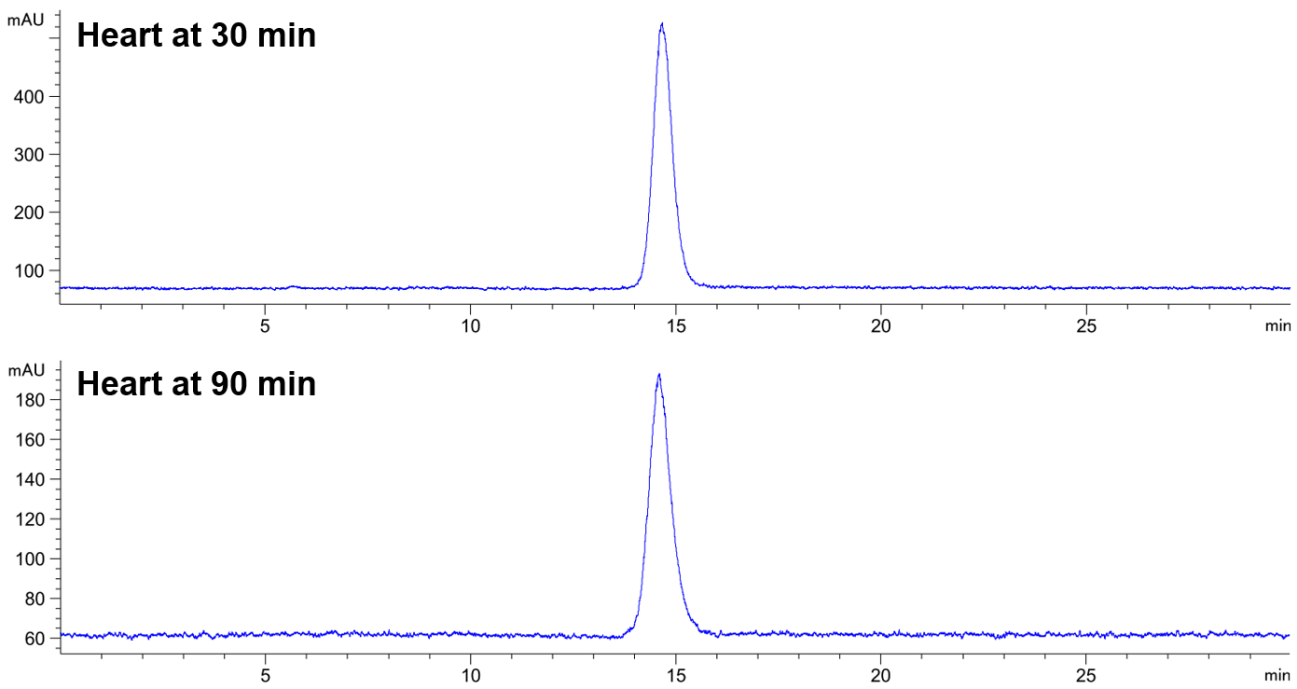
**

**c) Lung at 30 and 90 min**

**
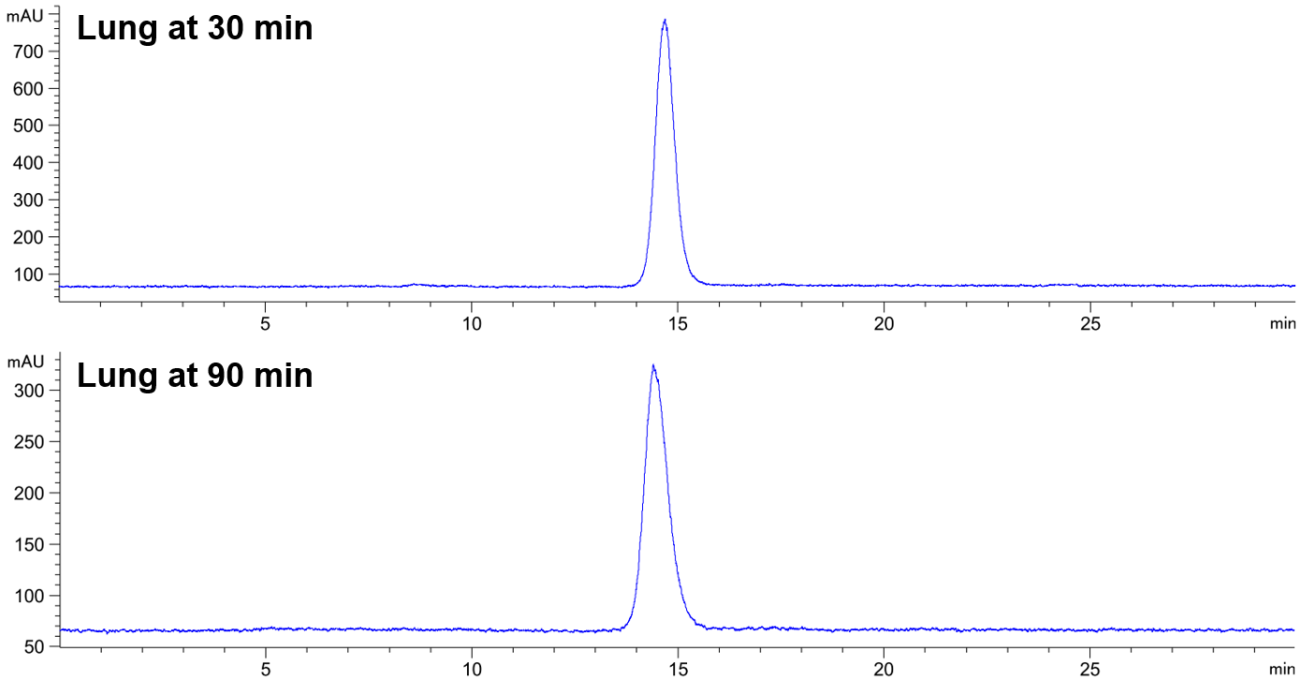
**

**d) Kidney at 30 and 90 min**

**
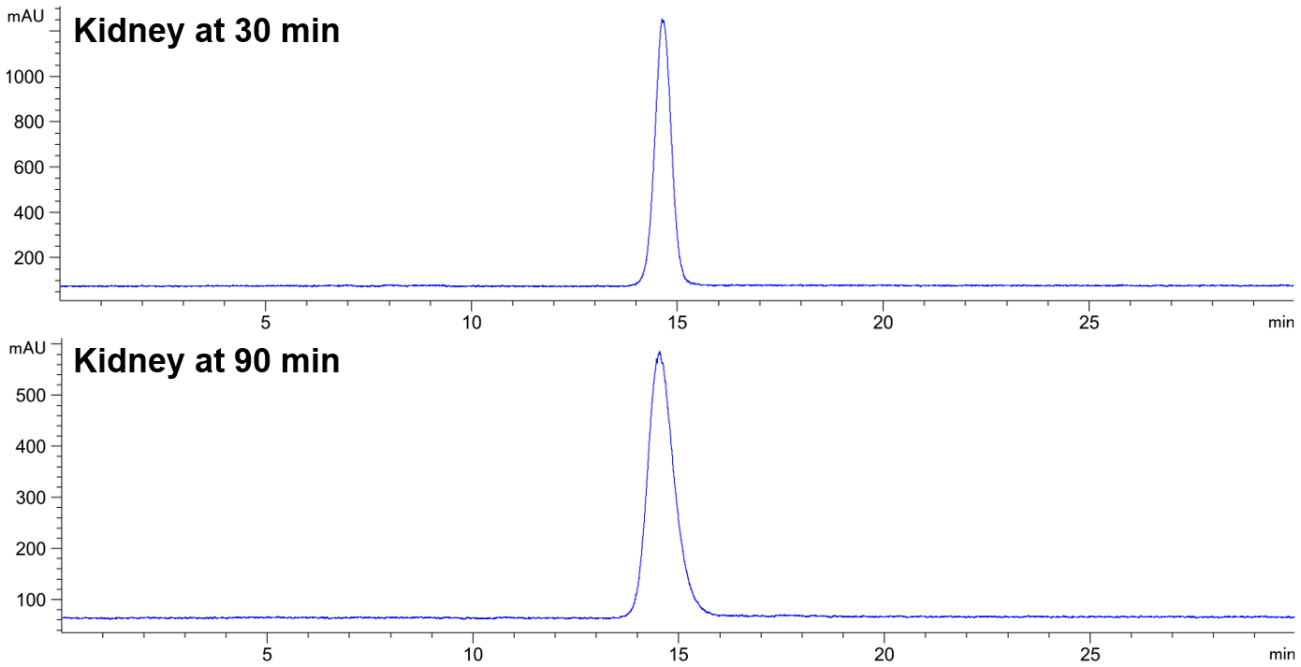
**

**e) Liver at 30 and 90 min**

**
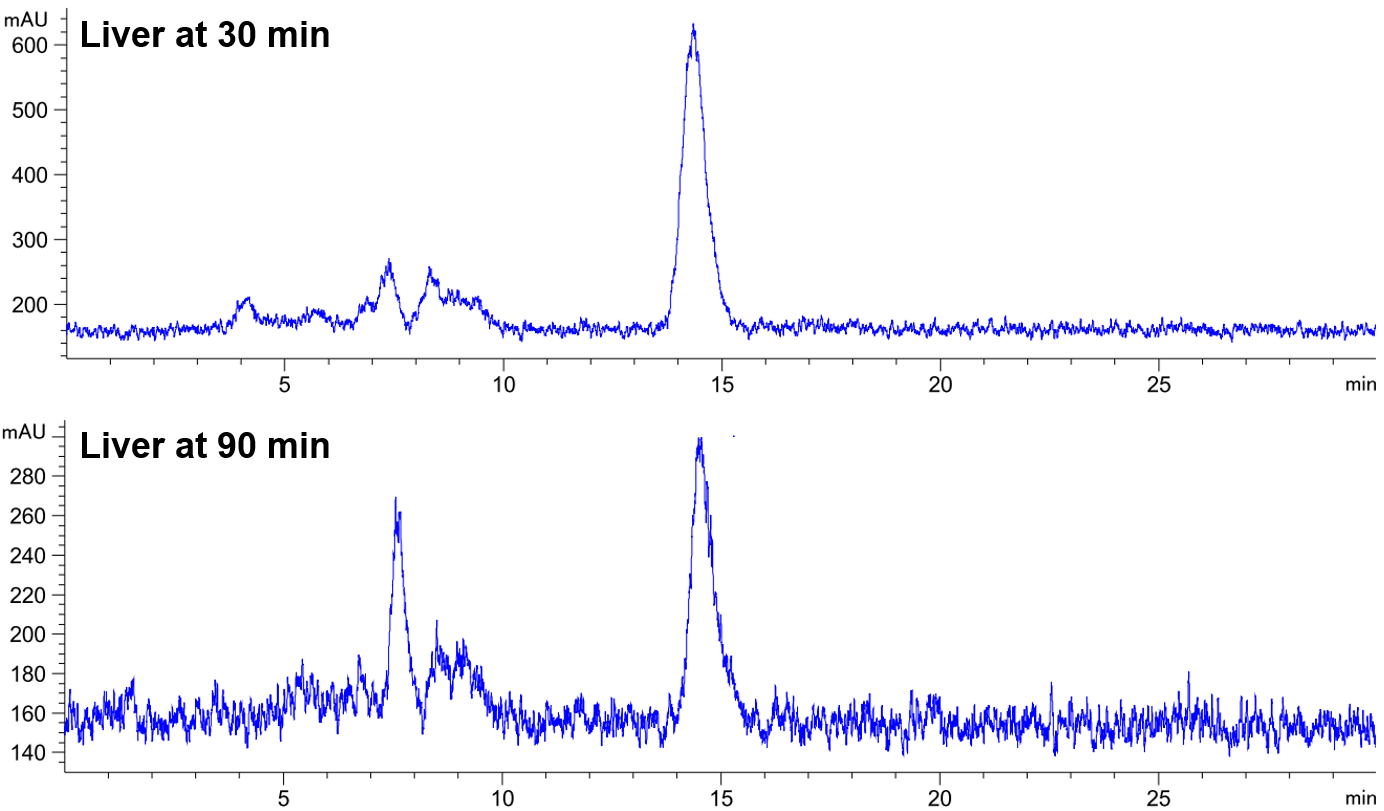
**

**f) Plasma at 30 and 90 min**

**
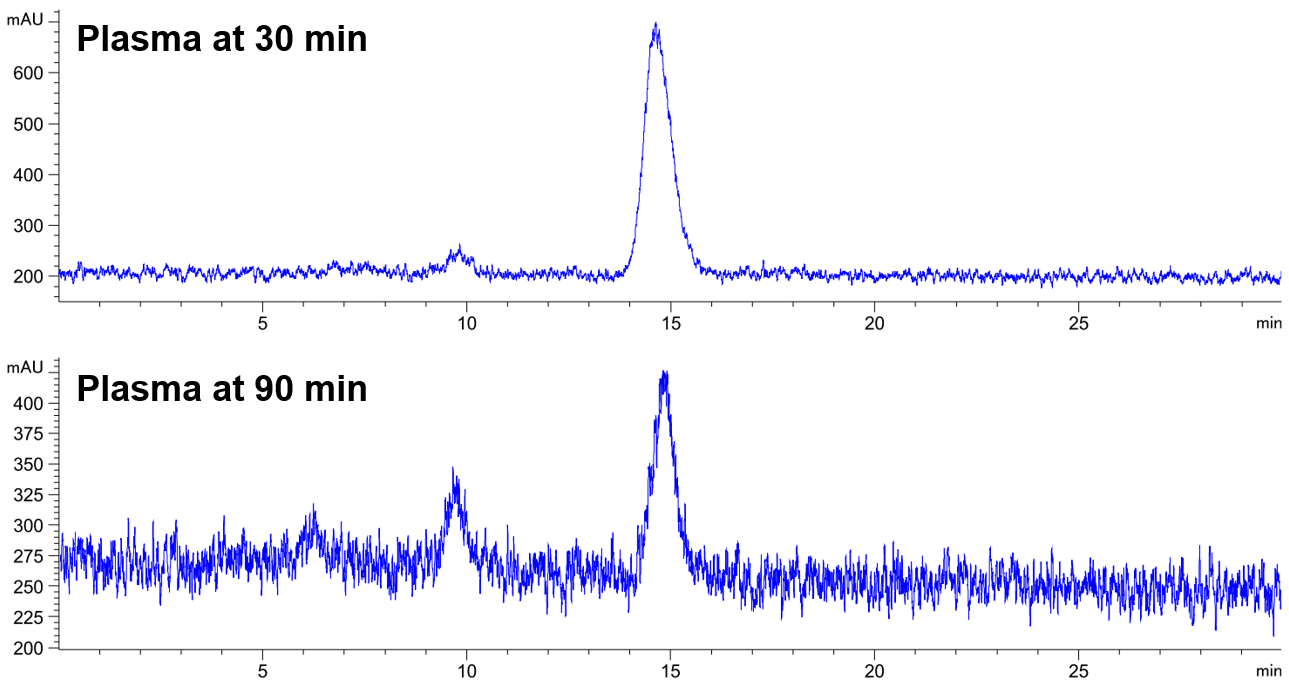
**

**Fig. S9** Autoradiography Images of [^18^F]BS224 Binding in MCAO Rat Brain Slices


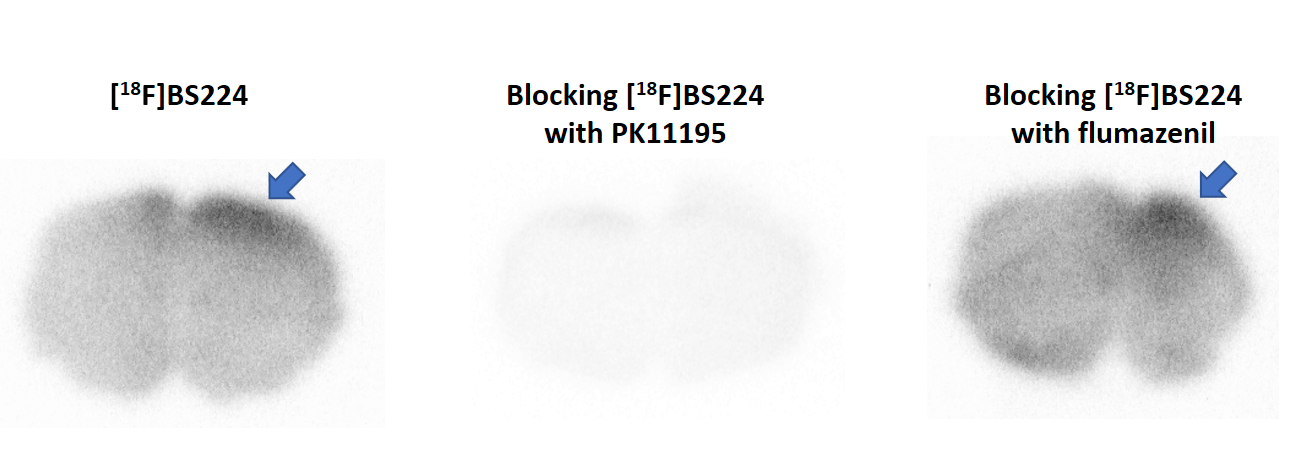


**The detailed procedure:** Brain section slides were incubated with 20 μCi of [^18^F]BS224 for 1 h at room temperature. For blocking study, PK 11195 (1 nmol) or flumazenil (1 nmol) was co-treated with [^18^F]BS224. Each slide was exposed to an imaging plate overnight. Arrow; the ischemia lesion.

**TABLE S1.** Log D Measurement of [^18^F]BS224

| Radioactivity (cpm) | 1 | 2 | 3 | 4 | 5 |
| --- | --- | --- | --- | --- | --- |
| octanol | 2076658.9 | 2156715.2 | 2220311.9 | 2183910.7 | 2165939.6 |
| PBS | 3478.7 | 3801 | 4084.9 | 3278.9 | 3311.2 |
|  | | | | | |
| Octanol/PBS | 596.964067 | 567.407314 | 543.54131 | 666.049803 | 654.1252718 |
| Log D | 2.77594819 | 2.75389493 | 2.7352326 | 2.8235067 | 2.815660928 |

Average of Log D = 2.78 ± 0.04, n = 5

**TABLE S2.** Measurement of Plasma Free Fraction for [^18^F]BS224

|  | Radioactivity (cpm) | | | | | | |
| --- | --- | --- | --- | --- | --- | --- | --- |
|  | in saline | | | in rat plasma | | in human plasma | |
|  | AS_total_ | AS_free_ | AP_total_ | | AP_free_ | AS_total_ | AS_free_ |
| 1 | 26346.8 | 23868.4 | 24296.3 | | 1412.5 | 35667.5 | 1361.8 |
| 2 | 25636.7 | 23073.1 | 23742 | | 1323.6 | 32919 | 1350.1 |
| 3 | 27040.8 | 25037.2 | 25561.2 | | 1436.7 | 31937.3 | 1392.4 |
| 4 | 41977.4 | 38555.5 | 27545.5 | | 1621.9 | 35394.7 | 1633.5 |
| 5 | 30210.9 | 27354.2 | 24709.9 | | 1553.6 | 36375.4 | 1899.1 |

**TABLE S3.** Estimated Human Absorbed Doses for Organs (µGy/MBq) of [^18^F]BS224 from Normal Mouse Data

| Target Organs | Estimated Human Absorbed Dose (µGy/MBq) |
| --- | --- |
| Adrenals | 09.66 ± 2.52 |
| Brain | 04.87 ± 0.90 |
| Breast | 02.28 ± 0.60 |
| Colon wall | 02.79 ± 0.55 |
| Endosteum (bone surface) | 00.91 ± 0.19 |
| ET region | 00.75 ± 0.14 |
| Eye lenses | 00.52 ± 0.09 |
| Gallbladder wall | 14.98 ± 3.89 |
| Heart wall | 10.54 ± 3.14 |
| Kidneys | 26.23 ± 8.91 |
| Liver | 28.88 ± 7.70 |
| Lung | 07.99 ± 2.42 |
| Lymphatic nodes | 02.60 ± 0.55 |
| Muscle | 00.77 ± 0.17 |
| Oesophagus | 03.90 ± 1.04 |
| Oral mucosa | 00.50 ± 0.09 |
| Pancreas | 07.79 ± 1.88 |
| Prostate | 00.43 ± 0.05 |
| Red (active) bone marrow | 02.01 ± 0.45 |
| Salivary glands | 00.49 ± 0.09 |
| Skin | 00.54 ± 0.12 |
| Small intestine wall | 07.30 ± 0.29 |
| Spleen | 02.90 ± 0.71 |
| Stomach wall | 09.06 ± 3.12 |
| Testes | 00.06 ± 0.00 |
| Thymus | 01.88 ± 0.52 |
| Thyroid | 00.95 ± 0.25 |
| Urinary bladder wall | 09.46 ± 2.73 |
| Effective dose ($\mu$Sv/MBq) | 05.18$\pm$1.03 |

Data are means ± SEM of three separate experiments.

**References**

S1. Jaremko L, Jaremko M, Giller K, et al. Structure of the Mitochondrial Translocator Protein in Complex with a Diagnostic Ligand. *Science.* 2014;343;1363-1366.

S2. Jaremko M, Jaremko L, Giller K, et al. Structural Integrity of the A147T Polymorph of Mammalian TSPO. *Chembiochem*. 2015;16:1483-1489.

S3. Schrödinger Release 2018-4: Prime, Schrödinger, LLC, New York, NY, 2018.

S4. Schrödinger Release 2018-4: Protein Preparation Wizard; Epik, Schrödinger, LLC, New York, NY, 2016; Impact, Schrödinger, LLC, New York, NY, 2016; Prime, Schrödinger, LLC, New York, NY, 2018.

S5. Schrödinger Release 2018-4: LigPrep, Schrödinger, LLC, New York, NY, 2018.

S6. Friesner RA, Murphy RB, Repasky MP, et al. Extra Precision Glide:  Docking and Scoring Incorporating a Model of Hydrophobic Enclosure for Protein−Ligand Complexes. *J Med Chem*. 2006;49:6177-6196.

S7. Schrödinger Release 2018-4: Glide, Schrödinger, LLC, New York, NY, 2018.

S8. Banks JL, Beard HS, Cao Y, et. al. Integrated Modeling Program, Applied Chemical Theory (IMPACT). *J Comp Chem*. 2005;26:1752-1780.

S9. Cristian CC, Evgueni S, Adriana G, Min LP and Jogeshwar M. Evaluation of [^18^F]Mefway Biodistribution and Dosimetry Based on Whole-Body PET Imaging of Mice. *Mol Imaging Biol.* 2013;15:1-17.

S10. Andersson M, Johansson L, Eckerman K and Mattsson S. IDAC-Dose 2.1, an internal dosimetry program for diagnostic nuclear medicine based on the ICRP adult reference voxel phantoms. *EJNMMI Res.* 2017;7:88.

S11. Moon BS, Kim BS, Park C, Jung JH, Lee YW, Lee H-Y, et al. [^18^F]Fluoromethyl-PBR28 as a Potential Radiotracer for TSPO: Preclinical Comparison with [^11^C]PBR28 in a Rat Model of Neuroinflammation. *Bioconjugate Chem.* 2014;25:442-450.

S12. Park SI, Jang DK, Han YM, Sunwoo YY, Park MS, Chung YA, et al. Effect of Combination Therapy with Sodium Ozagrel and Panax Ginseng on Transient Cerebral Ischemia Model in Rats. *J Biomed Biotechnol.* 2010;2010:893401.
